# Genome-wide association study of MRI markers of cerebral small vessel disease in 42,310 participants

**DOI:** 10.1038/s41467-020-15932-3

**Published:** 2020-05-01

**Authors:** Elodie Persyn, Ken B. Hanscombe, Joanna M. M. Howson, Cathryn M. Lewis, Matthew Traylor, Hugh S. Markus

**Affiliations:** 1 Department of Medical and Molecular Genetics, King’s College London, London, UK; 2 BHF, Cardiovascular Epidemiology Unit, Department of Public Health and Primary Care, University of Cambridge, Cambridge, UK; 3 Novo Nordisk Research Centre Oxford, Innovation Building, Old Road Campus, Roosevelt Drive, Oxford, UK; 4 Social, Genetic and Developmental Psychiatry Centre, King’s College London, London, UK; 5 Stroke Research Group, Department of Clinical Neurosciences, University of Cambridge, Cambridge, UK; 6 William Harvey Research Institute, Barts and The London School of Medicine and Dentistry, Queen Mary University of London, London, UK

**Keywords:** Genome-wide association studies, Genetics of the nervous system, Cerebrovascular disorders

## Abstract

Cerebral small vessel disease is a major cause of stroke and dementia, but its genetic basis is incompletely understood. We perform a genetic study of three MRI markers of the disease in UK Biobank imaging data and other sources: white matter hyperintensities (N = 42,310), fractional anisotropy (N = 17,663) and mean diffusivity (N = 17,467). Our aim is to better understand the disease pathophysiology. Across the three traits, we identify 31 loci, of which 21 were previously unreported. We perform a transcriptome-wide association study to identify associations with gene expression in relevant tissues, identifying 66 associated genes across the three traits. This genetic study provides insights into the understanding of the biological mechanisms underlying small vessel disease.

## Abstract (web summary)

Cerebral small vessel disease (CSVD) is a major cause of stroke and associated with structural changes of the brain. Here, Persyn et al. perform genome-wide association studies for magnetic resonance imaging (MRI) markers of CSVD, explore genetic correlations and prioritize candidate genes.

## Introduction

Cerebral small vessel disease (CSVD) causes a quarter of all strokes and is the most common pathology underlying vascular dementia^1^. Radiological markers include lacunar infarcts, white matter hyperintensities (WMH) and cerebral microbleeds. Despite its importance there is limited understanding of the pathogenesis and this is reflected in a lack of specific treatments for the disease. A number of arterial pathologies have been described including focal atheroma and diffuse arteriosclerosis. Brain parenchymal lesions include small infarcts, as well as regions of more diffuse white matter damage with ischaemic demyelination, axonal loss and gliosis, corresponding to WMH seen on T2-weighted magnetic resonance imaging (MRI). WMH themselves increase with age, and are associated with both stroke and dementia risk^2^. Studying MRI markers of CSVD such as WMH may provide important insights into SVD pathogenesis, by allowing asymptomatic disease to be studied in large community populations. Previous genome wide association studies (GWAS) have identified a number of loci associated with increased WMH risk, suggesting not only vascular but also glial and other neuronal cell genes may be involved^3–7^. However, such studies have been moderately powered. GWAS in other complex diseases including stroke^8^ have demonstrated the importance of very large sample sizes in identifying risk loci. The recent availability of data from the brain imaging substudy in UK Biobank offers an opportunity to greatly expand the sample size in which to explore the genetic basis of WMH and CSVD.

Previous GWAS studies of MRI marker of CSVD have largely focused on WMH. Diffusion tensor imaging (DTI) also measures white matter damage, but is likely to be more sensitive to disruption of normal function and structure rather than WMH which measure pathology alone. It allows estimation of mean diffusivity (MD) and fractional anisotropy (FA). MD looks at the diffusion of water molecules and is sensitive to diffuse white matter injury. FA measures the directionality of diffusion and is a marker of the integrity of white matter tracts. Previous studies have shown DTI parameters are abnormal throughout the white matter in CSVD, and not only within WMH, and are stronger predictors of dementia in CSVD than WMH^9,10^. DTI measures therefore might provide a more sensitive phenotype to identify CSVD risk genes. To date there has only been a single GWAS using DTI which identified a single locus^5^. We hypothesize that GWAS of DTI parameters might identify additional genetic loci, reflecting abnormalities in normal white matter structures occurring with CSVD. We also compare genetic associations between WMH and the DTI biomarkers, FA, and MD.

Using UK Biobank^11,12^, the Cohorts for Heart and Aging Research in Genomic Epidemiology (CHARGE) consortium^4,13^ and a WMH study in stroke patients^7^, we perform a GWAS of WMH in 42,310 individuals. Within UK Biobank, we perform a GWAS of DTI markers of white matter integrity. In addition to identifying genetic variants associated with the individual MRI markers of CSVD, we aim to identify genetic sharing between the different CSVD markers, and with other traits including common cardiovascular risk factors. Using external expression data, we also perform a transcriptome-wide association study (TWAS) to prioritize and identify new candidate genes for CSVD^14^.

From our genome-wide association studies, we identify 31 genetic loci, 21 of which have not been described in previous studies. We find genetic correlations with stroke, longevity, blood pressure, smoking and anthropometric traits. Transcriptome-wide association studies identify 66 candidate genes across the three imaging traits.

## Results

### Genome-wide association study

**Fig. 1:**
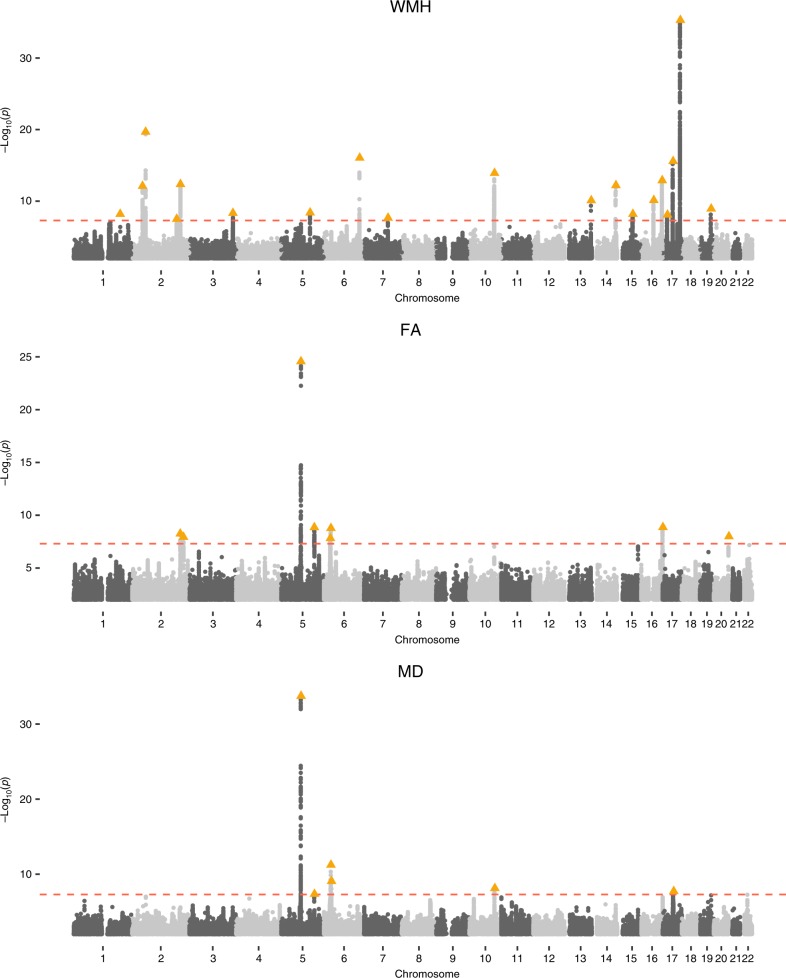
Genome-wide association Manhattan plots for WMH, FA and MD. Manhattan plots are shown for each of the phenotypes: WMH (top), FA (middle), and MD (bottom).

We conducted a GWAS on WMH in 18,381 European individuals from UK Biobank. The results were meta-analyzed with GWAS results from the CHARGE and WMH-Stroke multi-ethnic studies, for a total of 42,310 individuals. The intercept from LD score regression (LDSC) analysis (intercept = 1.00) suggested no statistical inflation. The quantile-quantile plot is available in Supplementary Fig. 1. From the meta-analysis, we identified 19 independent loci (*r*^*2*^ < 0.1) significantly associated (*p* ≤ 5 × 10^−8^) with WMH, ten of which are previously unreported (Fig. 1, Supplementary Data 1). By linear regression, we found that these ten variants account for 0.69% of the WMH variance in UK Biobank (14,577 individuals with non missing genotypes), while all 19 variants account for 1.79%. The regional plots for the top significant loci are available in Supplementary Fig. 2. We also performed a meta-analysis restricted to Europeans and found very similar results as 86% of participants in the three studies altogether were European (see Supplementary Fig. 3) (Table 1).

**Table 1:** Top association SNPs for independent loci for WMH meta-analysis.

CHR:BP	rsID	A1/A2	A1_FREQ	WMH_P	FA_P	MD_P	HGNC genes	Novel
1:197499003	rs12120143	T/C	0.03	6.45 × 10^−09^	3.78 × 10^−02^	3.79 × 10^−01^	*DENND1B*	Yes
2:43118872	rs7566761	A/G	0.20	7.62 × 10^−13^	1.95 × 10^−01^	6.57 × 10^−01^	AC098824.6^a^	No
2:56128091	rs7596872	A/C	0.10	2.06 × 10^−20^	3.92 × 10^−01^	1.48 × 10^−02^	*EFEMP1*	No
2:188003118	rs17576323	C/T	0.20	3.15 × 10^−08^	3.38 × 10^−01^	6.92 × 10^−01^	AC007319.1	Yes
2:203916487	rs72934505	G/T	0.13	4.31 × 10^−13^	7.34 × 10^−08^	7.28 × 10^−05^	*ICA1L, WDR12, CARF, NBEAL1, CYP20A1*	No
3:183380035	rs830179	A/G	0.32	4.67 × 10^−09^	3.21 × 10^−01^	1.11 × 10^−03^	*KLHL24*	Yes
5:121510586	rs17148926	C/A	0.17	4.07 × 10^−09^	1.54 × 10^−03^	4.46 × 10^−05^	*CTC-441N14.4*	Yes
6:151016058	rs275350	C/G	0.41	8.83 × 10^−17^	3.96 × 10^−03^	4.54 × 10^−05^	*PLEKHG1*	No
7:100361391	rs3215395	ID/G	0.29	2.18 × 10^−08^	1.21 × 10^−02^	4.42 × 10^−03^	*ZAN*	Yes
10:105459116	rs4630220	A/G	0.29	1.21 × 10^−14^	2.88 × 10^−03^	3.32 × 10^−05^	*SH3PXD2A*	No
13:111040681	rs11838776	A/G	0.28	7.90 × 10^−11^	1.97 × 10^−01^	1.03 × 10^−02^	*COL4A2*	No
14:100581636	rs11160570	T/C	0.26	6.10 × 10^−13^	1.09 × 10^−02^	3.19 × 10^−05^	*EVL, DEGS2*	No
15:65326833	rs12906662	A/T	0.47	6.42 × 10^−09^	8.81 × 10^−01^	1.85 × 10^−01^	*MTFMT, SLC51B*	Yes
16:51451683	rs17616633	T/C	0.44	7.33 × 10^−11^	2.01 × 10^−01^	4.97 × 10^−02^	*RP11-437L7.1*^a^	Yes
16:87237568	rs12928520	T/C	0.44	1.26 × 10^−13^	8.18 × 10^−01^	2.10 × 10^−01^	*C16orf95*	Yes
17:19224397	rs6587216	G/C	0.19	8.01 × 10^−09^	1.72 × 10^−01^	1.57 × 10^−02^	*EPN2*	Yes
17:43128906	rs8071429	T/A	0.37	2.61 × 10^−16^	9.17 × 10^−05^	4.14 × 10^−06^	*DCAKD, NMT1*	No
17:73882148	rs7214628	G/A	0.19	4.99 × 10^−36^	1.06 × 10^−01^	1.75 × 10^−03^	*WBP2, TRIM47, TRIM65*	No
19:45411941	rs429358	C/T	0.15	1.15 × 10^−09^	1.87 × 10^−02^	6.92 × 10^−04^	*APOE*	Yes

*CHR:BP* chromosome and position in bp, *rsID* the SNP ID, *A1/A2*, tested and non-tested alleles (ID is for insertion/deletions), *A1_FREQ* the allele frequency of the tested allele in the UK Biobank population for WMH, *WMH_P, FA_P and MD_P* the *p*-values for WMH, FA and MD respectively, *HGNC genes* the nearest genes to the lead SNP and its proxies (*r*^*2* ^≥ 0.8), genes symbols are in italic to comply with the nomenclature, *Novel* this column indicated if the association has already been described in previous GWAS.

^a^The lead SNP and/or proxies lie in an intergenic region.

GWAS of DTI parameters (FA and MD) was only performed in UK Biobank, as we did not have DTI data for the other cohorts (*N* = 17,663 and 17,467 respectively). We reduced each set of FA and MD DTI imaging measures in 48 brain regions to the first principal component which accounted for 38% (FA) and 41% (MD) of the variance in these measures (Supplementary Table 1, Supplementary Fig. 4). Association results showed no statistical inflation (FA intercept: 1.01; MD intercept: 1.01) and identified eight independent loci for FA (seven previously unreported loci), and six for MD (five previously unreported loci) (Table 2, Supplementary Data 2–3, Supplementary Figs. 5–8). We further investigated the significance of the FA and MD top SNPs from each genome-wide significant locus in the 48 brain regions separately (Supplementary Fig. 9). Results show a mixed pattern with some associations being across most brain regions, while others are more specifically associated with specific brain regions. By analyzing the first principal component, we capture the global white matter DTI measure signal.

**Table 2:** Top association SNPs for independent loci for FA and MD GWAS.

CHR:BP	rsID	A1/A2	A1_FREQ	WMH_P	FA_P	MD_P	HGNC genes	Novel
2:203664929	rs76122535	G/C	0.13	2.68 × 10^−12^	5.57 × 10^−09^	4.02 × 10^−06^	*ICA1L, WDR12, CARF, NBEAL1*	Yes
2:217325317	rs34380167	ID/C	0.27	2.81 × 10^−02^	1.16 × 10^−08^	8.98 × 10^−05^	*SMARCAL1, RPL37A*	Yes
5:82862328	rs35544841	ID/G	0.20	6.89 × 10^−07^	2.72 × 10^−25^	1.80 × 10^−34^	*VCAN*	No
5:139719991	rs4150221	C/T	0.26	8.30 × 10^−01^	1.39 × 10^−09^	4.40 × 10^−08^	*HBEGF*	Yes
6:26979765	rs374598428	ID/C	0.14	2.78 × 10^−02^	1.52 × 10^−8^	2.01 × 10^−07^	*LINC00240, VN1R12P*	Yes
6:28719755	rs1233587^b^	T/A	0.30	1.36 × 10^−01^	1.67 × 10^−07^	5.75 × 10^−12^	*ZFP57*^a^	Yes
6:29155749	rs3129171^b^	A/G	0.24	6.65 × 10^−03^	1.67 × 10^−09^	3.79 × 10^−09^	*ZFP57*^a^*, OR2J2, OR2H4P, XXbac-BPG308J9.3*	Yes
6:31329092	rs7772614	A/C	0.38	1.93 × 10^−02^	3.54 × 10^−05^	8.44 × 10^−10^	*HLA-B, HLA-S*	Yes
10:105682296	rs11813268	T/C	0.15	6.17 × 10^−04^	5.62 × 10^−05^	7.31 × 10^−09^	*STN1*	Yes
16:89951460	rs112730611	T/C	0.17	1.27 × 10^−02^	1.36 × 10^−09^	3.86 × 10^−06^	*SPIRE2, TCF25*	Yes
17:44013964	rs55939347	ID/T	0.22	2.49 × 10^−04^	2.98 × 10^−04^	1.84 × 10^−08^	*LINC02210-CRHR1, MAPT-AS1, MAPT, KANSL1*	Yes
20:61154871	rs6062264	T/C	0.28	8.53 × 10^−02^	1.02 × 10^−08^	6.77 × 10^−02^	*MIR1-1HG*	Yes

*CHR:BP* chromosome and position in bp, *rsID* the SNP ID, *A1/A2*, tested and non-tested alleles (ID is for insertion/deletions), *A1_FREQ* the allele frequency of the tested allele in the UK Biobank population for WMH, *WMH_P, FA_P and MD_P* the *p*-values for WMH, FA and MD respectively, *HGNC genes* the nearest genes to the lead SNP and its proxies (*r*^*2* ^≥ 0.8), genes symbols are in italic to comply with the nomenclature, *Novel* this column indicated if the association has already been described in previous GWAS.

^a^The lead SNP and/or proxies lie in an intergenic region.

^b^Associated SNPs for different traits which are in LD: rs1233587/rs3129171 (*r*^2^ = 0.42).

**Fig. 2:**
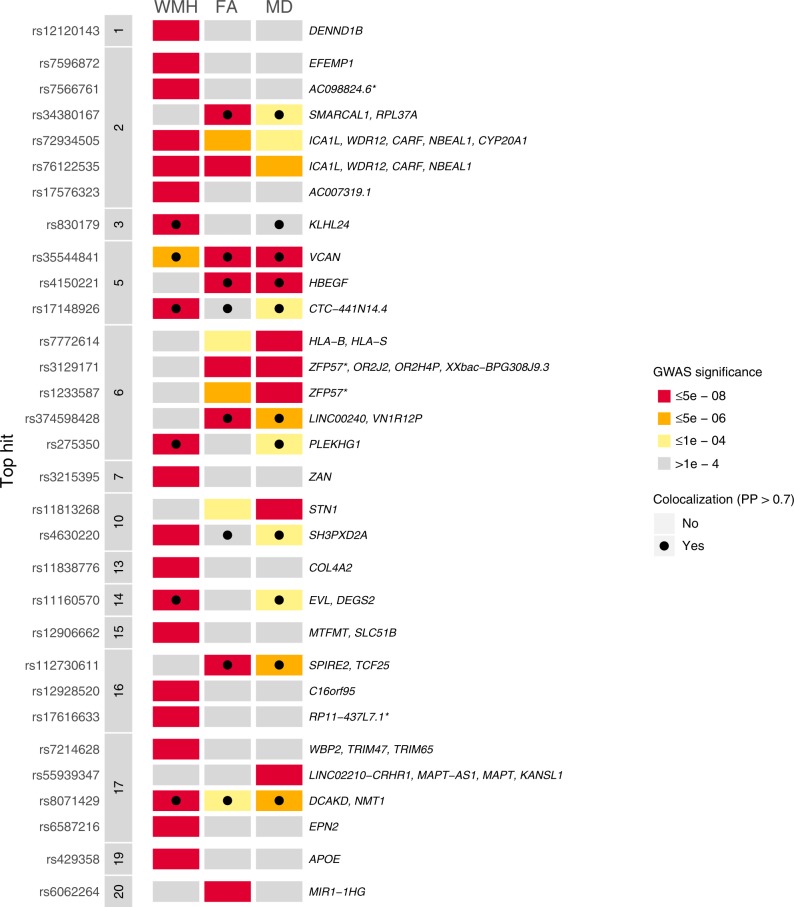
Comparison of association signals across WMH, FA and MD. Gene annotation was perfomed with PhenoScanner. ^*^The lead SNP and/or proxies lie in an intergenic region. PP is the posterior probability of colocalization.

Although a number of loci were shared between WMH and DTI markers, there were additional loci that appeared to be specifically associated with only WMH or DTI markers (Fig. 2). One significant locus in a high LD region on chromosome 2 was common to WMH and FA (respective lead SNPs: rs72934505, p_WMH_ = 4.31 × 10^−13^; rs76122535, p_FA_ = 5.57 × 10^−09^; *r*^2^ = 0.95). Three significant loci are common to FA and MD, two located on chromosome 5 (rs35544841, p_FA_ = 2.72 × 10^−25^, p_MD_ = 1.80 × 10^−34^; rs4150221, p_FA_ = 1.39 × 10^−09^, p_MD_ = 4.40 × 10^−08^), and one located on chromosome 6 (respective lead SNPs: rs3129171, p_FA_ = 1.67 × 10^−09^; rs1233587, p_MD_ = 5.75 × 10^−12^, *r*^*2*^ = 0.42).

### Pathway enrichment analysis

We performed a pathway enrichment analysis from our GWAS summary statistics using the Gene Ontology (GO) annotations^15,16^. We found 6, 0, and 4 GO terms which are significantly enriched for WMH, FA and MD respectively (false discovery rate (FDR) correction, adjusted α = 0.05, see Supplementary Table 2); there was no overlapping enriched GO term between WMH and MD results. Among these significant results, the GO term “D5 dopamine receptor b;inding” (*p* = 1.95 ×10^−06^) was the most significantly enriched molecular function term for WMH GWAS results, dopamine receptors being known to be involved in neurodegenerative diseases^17^. For MD, the GO term “voltage-gated calcium channel activity involved in AV node cell action potential” (*p* = 4.09 × 10^−07^) was the most significant one, concordant with a vascular mechanism underlying CSVD.

### Genetic sharing with MRI measures

In order to evaluate genetic sharing between the MRI markers of CSVD (WMH, MD, and FA), we calculated genome-wide genetic correlation using LDSC^18^ and performed colocalization analysis on the associated loci (Fig. 2)^19^.

SNP heritability estimates (*h*^2^) were 0.18 (se = 0.02) for WMH, 0.32 (se = 0.04) for FA and 0.27 (se = 0.04) for MD. There was strong evidence of genetic sharing between WMH, FA and MD with high genetic correlation estimates (WMH/FA: *r*_g_ = −0.25, se = 0.06, *p* = 3.2 × 10^−5^; WMH/MD: *r*_g_ = 0.41, se = 0.08, *p* = 8.7 × 10^−8^; FA/MD: *r*_g_ = −0.77, se = 0.03, *p* = 2.7 × 10^−114^).

**Fig. 3:**
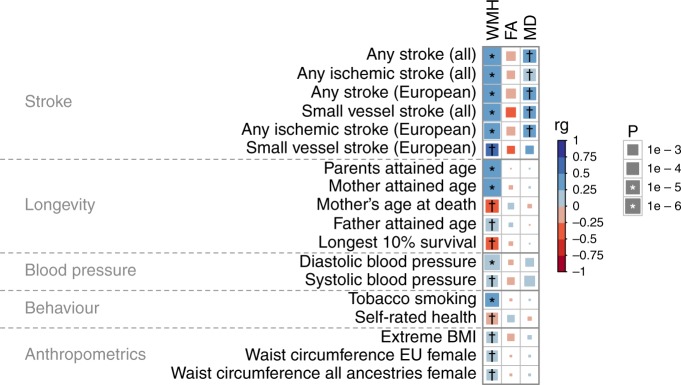
Genetic correlations between WMH, FA and MD traits and other traits. The symbols * and † indicate significant correlated traits by Bonferroni and FDR multiple testing correction respectively.

We also assessed the genetic correlation between our imaging biomarkers and traits from 479 available GWAS summary statistics (Supplementary Data 4). By applying FDR multiple testing correction for the 479×3 tests (p ≤ 7×10^-4^), we identified 23 significant genetic correlations with 18 traits (Fig. 3), which could be categorized into five groups (stroke, longevity, blood pressure, behavior and anthropometric traits).

We performed a sensitivity analysis based on the meta-analysis with only the UK Biobank and the CHARGE WMH studies, which did not include stroke patients. We found very similar genetic correlation results for the 18 traits which were significantly correlated with WMH (Supplementary Data 5).

With reference to common cardiovascular risk factors, significant associations were found for systolic and diastolic blood pressure, smoking, waist/hip ratio, BMI and alcohol use, but no association was found for diabetes or any lipid subfraction (cholesterol, triglyceride, HDL or LDL) (Supplementary Fig. 10 and Supplementary Data 4). We computed the variance explained by cardiovascular risk factors on WMH in UK Biobank using a linear regression and adjusting for the same covariates as in the GWAS. Although percentages are very small, these risk factors were highly significantly associated with WMH (Supplementary Table 3).

For each associated locus, we performed a multi-trait colocalization analysis^19^ including the three MRI markers of CSVD and stroke phenotypes from the MEGASTROKE study^8^. Twelve of the 31 MRI marker (WMH, FA, MD) associated loci colocalized with an alternate CSVD MRI marker and/or one or more stroke phenotypes from the MEGASTROKE study (with posterior probability > 0.7) (Supplementary Data 6). Of these twelve loci, eleven showed colocalization between at least two MRI biomarkers (Fig. 2), and three showed colocalization with at least one stroke phenotype. The WMH locus located on chromosome 5 (top SNP: rs17148926, candidate SNP in HyprColoc: rs17433120) was shared across WMH, FA, MD, any stroke (AS), any ischemic stroke (AIS) and small vessel stroke (SVS). The WMH locus on chromosome 13 (top SNP: rs11838776) was shared between WMH and SVS. These two loci were not significantly associated at GWAS significance with SVS in the MEGASTROKE study (rs17433120: *p* = 2.059 × 10^−07^, rs11838776: *p* = 1.086 × 10^−07^). The FA locus on chromosome 6 (top SNP: rs374598428, candidate SNP in HyprColoc: rs36022097) was shared between FA, MD and large artery stroke (LAS).

**Fig. 4:**
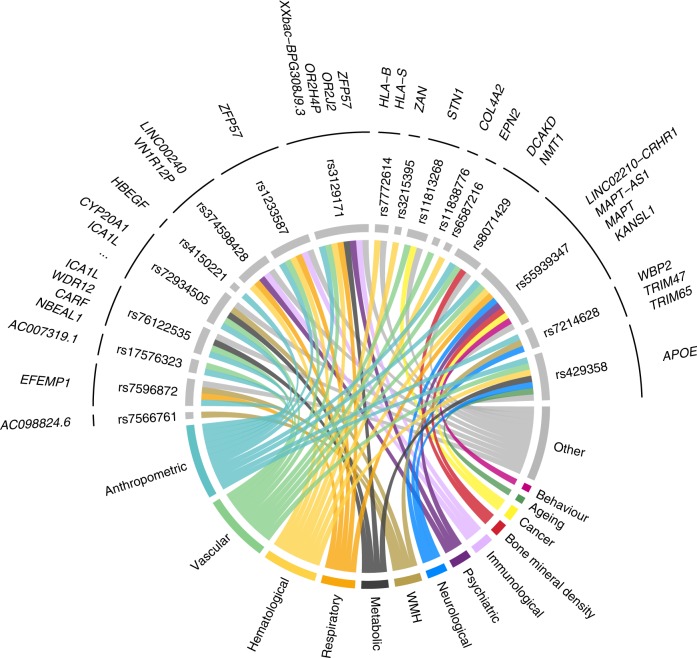
PhenoScanner disease/trait annotation for WMH, FA and MD loci. Only loci with a disease/trait annotation appear on this figure. For better visibility, we assigned each trait to one category with a specific color.

Pleiotropic effects of the 31 associated loci across WMH, FA and MD were evaluated with PhenoScanner to investigate locus-specific sharing across traits^20^. Eighteen were significantly associated with at least one additional trait (*p* ≤ 5 × 10^−8^) in the PhenoScanner database, primarily anthropometric, vascular, hematological, respiratory, and psychiatric traits (Fig. 4, Supplementary Data 7).

### Prioritizing candidate genes, tissues, and cell types

To identify genes whose expression is associated with risk of CSVD, we performed a TWAS integrating our GWAS results with expression quantitative locus (eQTL) data from CSVD-relevant tissues^14^. We focused our analyses on Genotype-Tissue Expression (GTEx)^21^ arterial and blood tissues and two larger eQTL studies from the CommonMind Consortium (CMC, brain)^22^ and Young Finns Study (YFS, blood)^23,24^. Arterial tissues from GTEx were more enriched by partitioned heritability analysis than the other tissues although there was not enough power to identify significantly enriched tissues for WMH and MD (Supplementary Figs. 11–13). Only the artery tibial tissue from GTEx was significantly enriched for FA. We also looked for cell-type enrichment by annotating each SNP to a brain cell type (pericytes, fibroblasts, microglia, smooth muscle cells, endothelial cells, oligodendrocytes, astrocytes) using mouse expression data^25^ and using MAGMA enrichment analysis. We did not find any significant result after correcting the significance threshold for the number of cell types (see Supplementary Table 4).

**Fig. 5:**
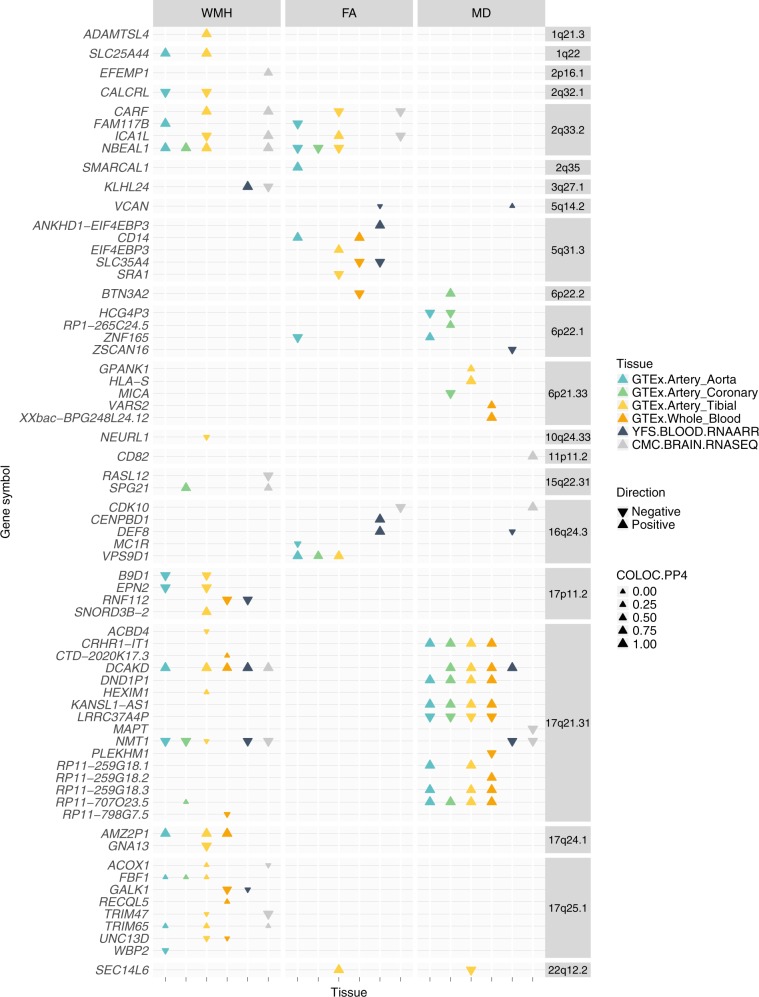
TWAS and COLOC results for WMH, FA and MD. COLOC.PP4 is the posterior probability of the hypothesis 4 in colocalization analysis, meaning there is association with the imaging biomarker trait and the gene expression and with one shared SNP.

From TWAS analysis of six tissues, we identified 33 significant gene expression/trait associations for WMH, 19 for FA and 27 for MD (at *p* ≤ 1.5 × 10^-6^, accounting for multiple testing correction) respectively (Fig. 5, Supplementary Data 8). In the 66 genes identified, 30 had no GWAS significant SNP (*p* ≤ 5 × 10^−8^) for WMH, FA or MD in the single-variant analysis implying that the gene-level TWAS analysis identifies associated loci which are not detected in the GWAS. Within each significant gene, TWAS results were mostly consistent across tissues, although a few genes had different direction of expression across tissues (*ICA1L*, *KLHL24*). We additionally performed a gene-set enrichment analysis from TWAS results for all genes, in each imaging trait for the six tissues. No significant GO terms were identified.

We further performed colocalization analysis for those genes whose expression was significantly associated with WMH, FA or MD. This complementary analysis aims to determine in which genes eQTL and MRI biomarker association signals colocalize as this can help prioritize candidate genes within a region. Of the 66 genes identified in the TWAS analyses, 48 of the MRI biomarkers and eQTL colocalized with a posterior probability ≥ 0.8 (COLOC hypothesis 4, H4) and so are consistent with the same underlying causal variant.

These results highlight regions and genes that are specific to each trait or contribute pairwise across traits. For example, on chromosome 17, *DCAKD* and *NMT1* imputed expression levels were significantly associated with WMH and MD, but not FA. Shared association for WMH and FA was detected on chromosome 2 (genes *CARF, FAM117B, ICA1L, NBEAL1*). We also found shared association for FA and MD on chromosomes 6 (gene *ZNA165*), 16 (gene *CDK10*) and 22 (gene *SEC14L6*).

## Discussion

We performed genome-wide association studies of CSVD related imaging traits in up to 42,310 individuals. We identified 33 associations overall, 19 with WMH (ten of which were previously unreported), eight with FA (seven previously unreported) and six with MD (five previously unreported). Our findings provide insights into the pathogenesis of CSVD, highlighting multiple pathways associated with disease risk. This study expands our previous study^7^ with the inclusion of the CHARGE summary statistics, and an additional 9,952 UK Biobank participants, increasing total sample size from 11,266 to 42,310.

To identify the genes and transcribed proteins influenced by the loci identified in our GWAS, we performed a transcriptome-wide association study, integrating mRNA expression data from relevant tissues. We coupled TWAS with colocalization analysis to identify trait-gene expression associations which were due to the same underlying causal variant. This approach enabled us to pinpoint the likely causal gene and tissue for a number of loci. They also suggest that some loci may act via increasing the small artery disease itself, while other may act via increasing brain responses to injury. The associations of elevated *ADAMTSL4* (ADAMTS-like 4), increased *SLC25A44* (Solute Carrier Family 25 Member 44), and decreased *CALCRL* (Calcitonin receptor-like) with WMH, and of *SEC14L6* (SEC14 Like Lipid Binding 6) with FA and MD, appear specific to the arteries, and therefore may increase risk via increasing the severity of the small vessel arteriopathy. Genes in the immediate vicinity of loci, or identified in the TWAS study, are associated with Mendelian vascular (*COL4A2, LOX, EPHB4, STN1*) or eye (*VCAN, ADAMTSL4, CRB1*) disease. One might expect these proteins to instead be involved in the core vascular processes underlying small vessel disease. Notably, four of these (*COL4A2, LOX, VCAN, ADAMTSL4)* are key extracellular matrix proteins, providing support for the hypothesis that the disruption of the cerebrovascular matrisome plays a central role in the pathogenesis of both monogenic and apparently sporadic CSVD^26^. In contrast for the chromosome 17q25 locus that has been described previously^3^, our analyses point to an association of decreased levels of *TRIM47* (Tripartite Motif Containing 47) in the brain with WMH. Similarly, among the loci which were not reported in previous publications, our analyses point to an association of *CD82* (Cluster of Differentiation 82) with FA and MD in the brain. One might expect these genes to be involved predominantly in the response of the brain parenchyma to ischemia.

Our findings also provide evidence to support the involvement of inflammatory and immunological processes in CSVD pathology. Most notably we identified associations of both FA and MD with variants in the human leukocyte antigen (HLA) region on chromosome 6, a gene complex encoding the cell-surface proteins involved in regulation of the immune system. For each of these traits, there were multiple independent loci (*r*^*2*^ < 0.1) spanning the extended HLA region reaching genome-wide significance. From these results alone we were not able to determine whether specific HLA alleles were associated: this should be the focus of future analyses. However this finding provides support for the hypothesis that inflammatory processes either in the vessels themselves, or at the blood-brain barrier, contribute to CSVD pathogenesis^27^.

The relationship between MRI markers of CSVD and dementia, particularly due to Alzheimer’s Disease (AD), remains controversial. WMH are a strong risk factor for dementia, and this is often assumed to be via ischemic damage contributing to both vascular and mixed dementia. However, a specific association of WMH with AD has also been proposed. WMH are increased in AD patients, and are an early core feature of autosomal dominant AD, occurring 6 years before symptom onset^28^. In this study we identified associations at genome-wide significance at the *APOE* locus, as well as with the chromosome 17 inversion which contains the *MAPT* gene, encoding microtubule associated protein tau, one of the key proteins involved in AD pathogenesis. Whether these associations reflect the fact the AD related changes influence WMH itself, or whether there is interplay between AD pathology and CSVD, as has been proposed^29,30^, is not clear from these data alone.

We also compared genetic associations between WMH and the DTI biomarkers, FA and MD. WMH represent pathological changes on MRI scans which on a population basis are usually caused by CSVD. DTI markers also measure white matter damage but are likely to be more sensitive to disruption of normal function and structure rather than measuring pathology alone. Our study performed comprehensive analysis of the genetic architecture of these different white matter markers. While there was significant genetic correlation between WMH and DTI markers, we also identified significant genetic differences. A number of loci were risk factors for both WMH and DTI markers, but others were specific to either WMH or DTI. Of note we found that three of the four loci which were selectively associated with DTI markers and colocalized between only FA and MD, overlap with genes that contain variants previously reported in GWAS for intelligence, cognition or schizophrenia (*HBEGF*^31,32^, *SMARCAL1*^33^, *VN1R12P*^31,34^). Two other GWAS loci specific to FA and/or MD, were also found associated with schizophrenia, autism (*OR2J2*^35,36^), intelligence and psychiatric measurements (*MAPT*^37–39^). We also found from the TWAS analyses genes in which variants were found associated autism, schizophrenia, neuroticism, depression, cognitive ability and intelligence (*BTN3A2*^40^, *CRHR1-IT1*^41^, *DND1P1*^41^, *KANSL1-AS1*^41^, *MAPT*, *MICA*^35^, *PLEKHM1*^38^, *SLC35A4*, *ZNF165*^35^) and they were also mainly specific to FA and MD. These findings suggest DTI measures represent a marker of alterations to normal brain networks, and that such networks may play in role in the genesis of psychiatric disorders.

Our TWAS study identified an association of decreased levels of Calcitonin receptor-like (*CALCRL*) with increased WMH. *CALCRL* is a protein which, when associated with *RAMP1*, produces the calcitonin gene related peptide (CGRP) receptor, or when associated with *RAMP2*, produces the adrenomedullin (ADM) receptor. CGRP and ADM are potent vasodilators with ameliorating effects on cardiovascular disease. There is evidence from mice that targeting the CGRP pathway could ameliorate cerebral ischemia^42,43^, and trials have investigated its influence on cerebral ischemia in postoperative aneurysmal subarachnoid hemorrhage^44^. Whether targeting CGRP or ADM could ameliorate the long-term ischemic changes underlying CSVD should be the subject of further study.

Our study has limitations. Participants were of predominantly European ancestry and our findings can therefore not be generalized to individuals of all ancestries. Our study included three sets of GWAS results for WMH—two were from population based studies while the third was from a cohort of stroke patients. The stroke group had more severe WMH. The genetic architecture of WMH in community populations and stroke patients appears to be similar^6^. However to explore whether inclusion of these stroke cases may have altered results we performed a sensitivity analysis excluding these cases, and very similar associations for the same 18 loci were found. We included all cases in the discovery cohort to increase power to identify new associations. We did not include a replication cohort. Elevated blood pressure is a significant risk factor for CSVD. We did not adjust for blood pressure in our analysis as this can result in biased estimates of genetic effects^45^, or worse can lead to false positive associations due to collider bias^46^. In TWAS analyses, we focused on artery and blood tissues from GTEx, blood from the YFS and brain tissue from the CMC study. More specific cell types in relation to CSVD pathogenesis would also greatly help in the understanding of underlying biological processes. We did not include in our analyses the different brain tissue GTEx expression data as they were far from being enriched in the partitioned heritability analysis we performed. Also, it is important to not overinterpret TWAS results in terms of causality as imputed gene expression might be associated with non-causal SNPs; for this reason, we conducted colocalization analyses to help in prioritizing these genes. In following up GWAS results, we focused primarily on using TWAS results to highlight potentially implicated genes. It should not be forgotten that other mechanisms, such as alterations in protein function, splicing, and various epigenetic processes could also confer disease risk. Indeed a common missense variant in *TRIM47* (p.Arg187Trp) might be the underlying risk mechanism at the 17q25 locus.

In summary we identified 33 associations (31 loci) with CSVD-related imaging traits. Our findings increase the knowledge of the genetic basis of CSVD-related imaging traits, showing that certain loci confer risk of both WMH and DTI measures, while others are related to one or the other. Our results highlight the involvement of the cerebrovascular matrisome in CSVD, and provide further evidence of the involvement of inflammatory mechanisms.

## Methods

### UK Biobank study population

UK Biobank is a major data health resource including ~500,000 participants from across the UK, aged between 40 and 69 years at recruitment^47^. The UK Biobank includes clinical and phenotypic information for a broad range of traits and includes MRI imaging data on a subset of participants. In this study, we used the UK Biobank imaging data on ~20,000 individuals released in October 2018^11,12^. MRI was performed on two identical Siemens Skyra 3.0 T scanners (Siemens Medical Solutions, Germany), running VD13A SP4, with a standard Siemens 32-channel RF receiver head coil. Identical acquisition parameters and careful quality control (QC) was used for all scans. We selected individuals described for three phenotypes all of which variables already obtained from the UK Biobank MRI data by the central MRI analysis centre in Oxford (1) total volume of WMH (from T1 and T2_FLAIR images) WMH (field 25781), (2) FA (fields 25056-25103) and (3) MD (fields 25104-25151) (see Supplementary Data 9 for field description). Individuals diagnosed with stroke, or with other major CNS disease which could be associated with white matter damage (e.g., multiple sclerosis, Parkinson disease, dementia or any other CNS neurodegenerative condition) were excluded from the analysis (see Supplementary Table 5 for removed codes description).

### Additional WMH cohorts

We obtained WMH summary statistic results from the CHARGE consortium through the database of Genotypes and Phenotypes (dbGaP) (study: phs000930.v6.p1). This multi-ethnic study included 21,079 individuals free of dementia and stroke of European (*N* = 17,936), African (*N* = 1943), Hispanic (*N* = 795), and Asian (*N* = 405) ancestry^4^.

We also obtained WMH GWAS summary statistics from a study in 2850 ischemic stroke patients^7^, including 2694 and 156 individuals of European and African ancestry respectively. In the original study, individuals with any monogenic cause of stroke, vasculitis, or any other non-ischemic cause of WMH such as demyelinating and mitochondrial disorders were excluded from this dataset.

### Image analysis assessment

We used WMH, FA and MD imaging-derived phenotypes generated by an image-processing pipeline developed and run on behalf of UK Biobank (https://biobank.ctsu.ox.ac.uk/crystal/crystal/docs/brain_mri.pdf)^11,12^. WMH trait was log-transformed and normalized for brain volume (field 25009). For each biomarker, outliers outside the ± 6 s.d. range were removed.

DTI measures were available as part of the UK Biobank central analysis for 48 individual white matter regions. To obtain a single global measure of global white matter FA and MD from the DTI images, principal component analysis (PCA) was performed on the FA and MD measures of each of the 48 different brain tracts analyzed, using FactoMineR^48^, as a dimension reduction method. The first principal component for FA and MD was used for association analysis (see Supplementary Table 1 for more details about the PCA analysis). In addition, as a secondary analysis, we performed analysis for each brain region independently.

### Genetic data and QC

We used genotype data imputed to the Haplotype Reference Consortium panel and released by UK Biobank in June 2017. Imputation and QC procedures from the UK Biobank study are described in^47^. From the UK Biobank sample QC description, we excluded (1) related individuals with a KING kinship coefficient ≥ 0.0884 (to keep only one individual per group of up to second-degree relationships)^49^, (2) individuals with mismatch between genotype and reported sex, (3) outliers in terms of heterozygosity and genotype missingness (individuals with a missing rate > 5%), (4) individuals not contained in a homogeneous cluster of European ancestries based on PCA and *k*-mean clustering (*k* = 4) on the two first PCs. After this filtering, we performed further PCA on non-correlated common SNPs (*r*^*2*^ < 0.2 and minor allele frequency ≥ 5% (MAF)) with PLINK 2.0 software (www.cog-genomics.org/plink/2.0/)^50–52^. Population outliers were iteratively excluded if they were outside the ±8 s.d. range for the first 10 PCs (see Supplementary Table 6 for numbers of participants removed at each QC step, and Supplementary Figs. 14–16 for PCA plots for genetic population structure). For SNP QC, we removed SNPs with an imputation INFO score <0.5, a MAF < 1% or a Hardy-Weinberg disequilibrium *p*-value ≤ 1 × 10^−10^.

### Genome-wide association study

Association analysis was performed using linear regression on WMH (*N* = 18,381), FA (*N* = 17,663) and MD (*N* = 17,467) for ~9.7 million SNPs with PLINK 2.0 (www.cog-genomics.org/plink/2.0/)^50,51^ based on genotype dosages from imputation. Covariates included (1) age at MRI (derived from UK Biobank fields 34, 52 and 53), (2) sex (field 31), (3) genotyping array (field 22000; Affymetrix UK BiLEVE or UK Biobank Axiom Array), (4) the UK Biobank assessment centre (field 54; Cheadle or Newcastle), (5) the first 10 PCs, (6) MRI head motion indicators which are mean tfMRI head motion (field 25742) and Mean rfMRI head motion (field 25741) (see Supplementary Tables 7–9 for further details). Missing values for MRI head motion indicators were imputed using the R package mice with the predictive mean matching method^53^ based on all covariates. A meta-analysis was performed of the UK Biobank WMH GWAS results with GWAS results from two multi-ethnic studies described above, the CHARGE study (*N* = 21,079)^4^ and the WMH study in stroke patients (WMH-Stroke; *N* = 2850)^7^, giving a total of 42,310 individuals. As beta values were not available in the CHARGE study, a Z-score based meta-analysis was performed using METAL^54^. Genomic inflation was assessed by using LDSC intercepts^55^. Genome-wide statistical significance was set as *P* ≤ 5 × 10^−8^. Significant independent loci were defined as clumped significant association results with PLINK (--clump-kb 1000 --clump-r2 0.1), i.e., groups of SNPs in LD (*r*^2 ^≥ 0.1) in 1000 kb windows and represented by the most significant SNP, and merged across overlapping 250 kb neighboring genetic windows. Regional association plots, showing LD between independent top association SNPs and 250 kb neighboring SNPs were constructed based on a subset of 1000 UK Biobank individuals as a reference LD panel.

### Gene-set enrichment analysis from GWAS results

From GWAS summary statistics, we conducted gene based analysis and gene set enrichment analysis using MAGMA program^56,57^. Genes boundaries were defined using NCBI 37.3 gene annotations (https://ctg.cncr.nl/software/MAGMA/aux_files/NCBI37.3.zip). SNPs were mapped to genes and within 10 kb flanking regions. Gene-based association analysis was then performed using summary statistics from all GWAS tested SNPs for WMH, FA and MD. We used the European ancestry UK Biobank reference dataset to take into account the LD structure in the gene-based association testing. We conducted gene-set enrichment analysis based on GO terms and mouse brain cell-type expression data^25^. For the first analysis, we defined gene sets with GO annotations (http://geneontology.org/, May 2019 release)^15,16^, kept gene sets with more than three genes and applied the competitive enrichment testing. Results were reported according to the three main categories of GO terms called biological process (GO:0008150), molecular function (GO:0003674) and cellular component (GO:0005575). For the second analysis, we used mouse brain expression data and defined cell types gene sets as the top 100 or top 500 most differentially expressed genes between the cell types versus all 15 cell types. We translated mouse gene names into the ortholog human gene names. We defined significantly enriched gene-sets by adjusting *p*-values with the Benjamini-Hochberg FDR multiple testing correction and setting a 5% threshold.

### Assessment of pleiotropy

We annotated association results with PhenoScanner (R Package phenoscanner v1.0)^20^. For each independent locus, we queried the top significant SNP and its proxies (SNPs with *r*^2^ > 0.8) using our European ancestry UK Biobank reference dataset (sampling of 1000 individuals from our population study). We retained PhenoScanner GWAS association results with a *p*-value < 5 × 10^−8^.

### Multiple trait colocalization analysis

We performed colocalization analysis to identify shared genetic loci between WMH, FA and MD and stroke traits with the HyPrColoc program^19^. We downloaded GWAS summary statistics for stroke traits from the MEGASTROKE study, a large GWAS of stroke, and its major subtypes^8^. Genetic loci in the colocalization analysis were defined as the top hit per independent associated locus with +/−500 kb flanking regions. We identified colocalized traits by setting a posterior probability threshold of 0.7. We retained in the results combinations of traits containing the trait the genetic locus was associated with.

### Heritability and genetic correlation

For WMH, FA and MD traits, SNP heritability and genetic correlations were assessed using LDSC^18,55^. For QC, we filtered well-imputed SNPs by using the HapMap3 LD reference in the European population^58^. We excluded SNPs from the major histocompatibility complex as they display high LD and could bias the LDSC analysis results. Genetic correlations were assessed between the WMH, FA and MD traits, and also with 479 phenotypes from open source GWAS summary statistics data from (1) the Navigome online tool^59^, (2) a recent study on blood pressure^60^, (3) a recent study on AD^61^ and (4) the MEGASTROKE study^8^. We tested for statistical significance of the observed genetic correlation after applying both FDR (*q*-value < 0.05) and Bonferroni (*p* < 0.05/(479 × 3)) multiple testing correction methods.

We also partitioned the SNP heritability of WMH, FA and MD by functional category^62,63^ using the 44 tissues in the GTEx data^21^ and in astrocyte, neuron, and oligodendrocyte expression data^64^.

### Contribution of risk factors and genetic loci to WMH

We estimated the contribution of the top associated SNPs to WMH variance in UK Biobank by deriving the difference in coefficient of determination (*R*^2^) between the two nested linear regression models (including covariates with and without the top associated SNPs). We also estimated the contribution of vascular risk factors to WMH variance. The vascular risk factors we chose and the UK Biobank fields used to derive them are listed in Supplementary Table 3. For each of WMH and the vascular risk factors, we performed a regression model incorporating the same covariates as in the GWAS and derived the residuals (model: trait ~ covariates). Then we regressed residuals for each risk factor on WMH residuals (model: WMH residuals ~ risk factor residuals) and retrieved the adjusted *R*^*2*^.

### TWAS and colocalization analysis

We performed a TWAS with FUSION^14^, from gene expression models derived from the CMC^22^, YFS^23,24,65^, and GTEx v7 datasets^21^. The CMC gene expression tissues (labeled as CMC-brain) were collected from dorsolateral prefrontal cortex in individuals with schizophrenia or control individuals (*N* = 452). In the YFS study (labeled as YFS-whole blood), peripheral blood gene expression has been collected for 1650 participants (*N* = 1,264). Among the available GTEx tissues, we focused our TWAS analysis on aorta artery (*N* = 267), coronary artery (*N* = 152), tibial artery (*N* = 388) and whole blood (*N* = 369), based on the assumption that these tissues would be the most relevant for CSVD pathogenesis. Bonferroni correction for multiple testing was applied taking into account the total number of tested genes across the tissues. TWAS results were further investigated with colocalization analysis of eQTLs and GWAS signals with the R package COLOC^66^, to assess whether the observed eQTL and GWAS associations were consistent with a common shared association.

### Gene-set enrichment analysis from TWAS results

From all genes TWAS results, we conducted a gene-set enrichment analysis using the program TWAS-GSEA^67^ for GO terms (downloaded from MSigDB, February 2019 Gene Ontology release)^68,69^. TWAS-GSEA preforms first a fixed-effects linear regression on the model ${\mathrm{TWASpvalue}}_g = {\mathrm{GeneSet}}_g + {\mathrm{GeneLength}}_g + {\mathrm{eQTLnumber}}_g + \varepsilon _g$ with *g* as the gene index. In a second step, after FDR multiple testing correction, significant gene sets were tested by mixed linear regression taking into account the correlation between the gene expressions as a random effect. The gene expression correlation matrix was computed from predicted expression in 1000 Genomes European sub-population (*N* = 489). We performed this gene-set enrichment analysis for WMH, FA and MD and the six tissues we selected.

### Ethical considerations

This research has been conducted using the UK Biobank Resource under application number 36509. UK Biobank received ethical approval from the Research Ethics Committee (reference 16/NW/0274). CHARGE summary statistics were obtained through the dbGaP portal application number 19896 (study: phs000930.v6.p1). Summary statistics from the WMH study in stroke patients were obtained through agreement with the authors^7^. All studies obtained informed consent from all participants and got ethical approval from their local ethics committee; full ethical permissions of contributing studies have been previously published.

### Reporting summary

Further information on research design is available in the Nature Research Reporting Summary linked to this article.

## Supplementary information

Dataset 1

Dataset 2

Dataset 3

Dataset 4

Dataset 5

Dataset 6

Dataset 7

Dataset 8

Dataset 9

Supplementary Information

Reporting Summary

Description of Additional Supplementary Files

## Data Availability

This analysis used publicly available data from the UK Biobank (www.ukbiobank.ac.uk, field codes are described in the Supplementary Data 13 and the Supplementary Table 7), WMH stroke study (http://cerebrovascularportal.org/informational/downloads) and CHARGE (https://www.ncbi.nlm.nih.gov/gap/, we used data from the study phs000930.v6.p1, the currently available version is phs000930.v7.p1). The GWAS summary statistics from WMH, FA, and MD for the UK Biobank and stroke studies are available via the Cerebrovascular Disease Knowledge Portal (http://www.cerebrovascularportal.org/) Data Downloads page (http://www.kp4cd.org/dataset_downloads/stroke). We obtained the CHARGE summary statistic data directly from dbGaP. We are unable to make them available via the cerebrovascular disease portal due to dbGaP and CHARGE access regulations, and these can be obtained direct from dbGaP (https://www.ncbi.nlm.nih.gov/gap/). In our post-GWAS analyses, we used the Gene Ontology database (http://geneontology.org/), MAGMA software gene definitions (https://ctg.cncr.nl/software/magma), the PhenoScanner database (http://www.phenoscanner.medschl.cam.ac.uk/), LDSC LD scores (https://github.com/bulik/ldsc), GWAS summary statistics (the list of Pubmed IDs is provided in the Supplementary Data 5), FUSION software weights and reference LD (http://gusevlab.org/projects/fusion/), differential expression data in mouse brain cell types (http://betsholtzlab.org/VascularSingleCells/database.html).

## Code availability

All code used to perform the different analyses is available in https://github.com/elodiepersyn.
