# Supplementary material for: Genome-wide association study of MRI markers of cerebral small vessel disease in 42,310 participants
Source: Nat Commun. 2020 May 1;11:2175. doi: 10.1038/s41467-020-15932-3 (PMC7195435; doi:10.1038/s41467-020-15932-3)
Supplement: Supplementary file 10 — Supplementary Information [file 41467_2020_15932_MOESM10_ESM.pdf]

Genome-wide association study of MRI markers of cerebral small vessel disease in 42310 participants

Persyn et al.

Supplementary Figures

Supplementary Figure 1. QQ plot for WMH GWAS in UK Biobank. 4
Supplementary Figure 2. Regional plots of WMH significant association results. 14
Supplementary Figure 3. Comparison between European and trans-ethnic results for WMH reported top SNPs.. 15
Supplementary Figure 4. PCA correlation plots. 16
Supplementary Figure 5. QQ plot for FA GWAS in UK Biobank. 17
Supplementary Figure 6. QQ plot for MD GWAS in UK Biobank 18
Supplementary Figure 7. Regional plots of FA significant association results. 23
Supplementary Figure 8. Regional plots of MD significant association results. 27
Supplementary Figure 9. Association results in the different brain regions. 28
Supplementary Figure 10. Genetic correlation results with CSVD comorbidities. 29
Supplementary Figure 11. Tissue enrichment in WMH GWAS meta-analysis summary statistics. 30
Supplementary Figure 12. Tissue enrichment in FA GWAS summary statistics. 31
Supplementary Figure 13. Tissue enrichment in MD GWAS summary statistics. 32
Supplementary Figure 14. Graph of individuals for PCA on WMH dataset. 33
Supplementary Figure 15. Graph of individuals for PCA on FA dataset. 34
Supplementary Figure 16. Graph of individuals for PCA on MD dataset. 35

Supplementary Tables

Supplementary Table 1. PCA eigenvalues and explained percentages of variance for FA and MD measure derivation....36
Supplementary Table 2. GO enrichment analysis....37
Supplementary Table 3. Contribution of cardiovascular risk factors to WMH measure in UK Biobank population....38
Supplementary Table 4. Mouse brain cell-type enrichment analysis. 39
Supplementary Table 5. Removed field codes description....40
Supplementary Table 6. Sample quality control description....41
Supplementary Table 7. UK Biobank field codes to derive covariates. 42
Supplementary Table 8. Covariate description....43
Supplementary Table 9. Description of covariates in relation to WMH, FA and MD phenotypes 44

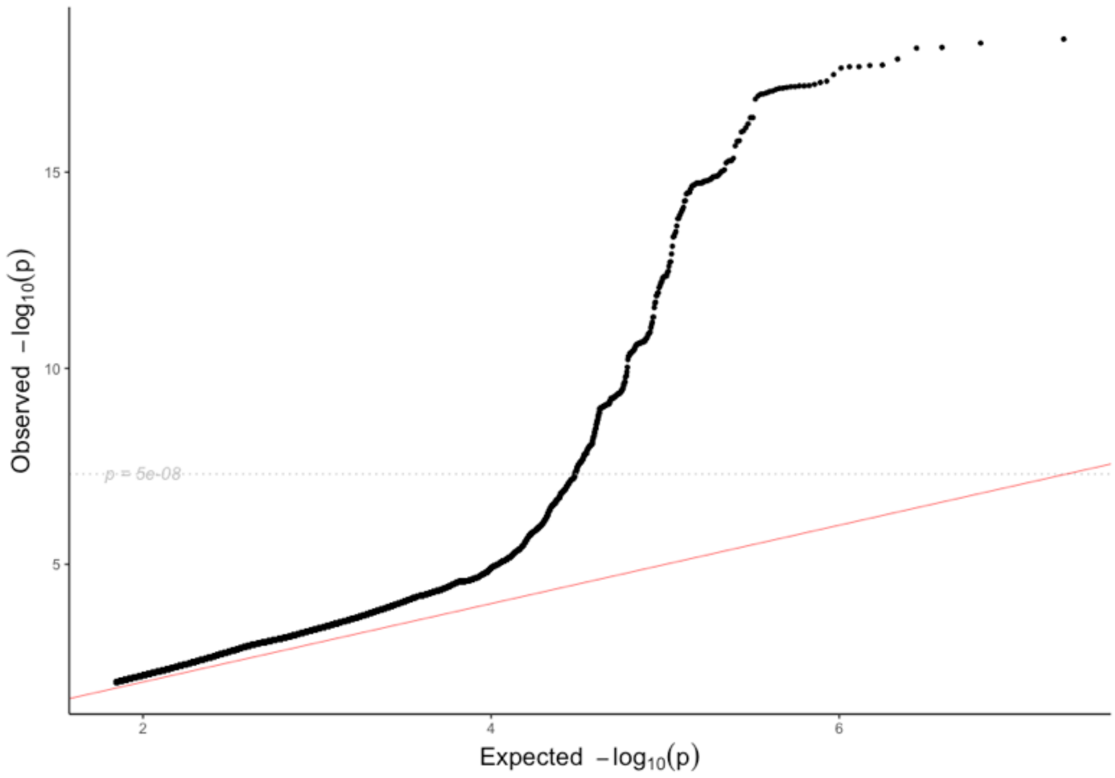

Supplementary Figure 1. QQ plot for WMH GWAS in UK Biobank.

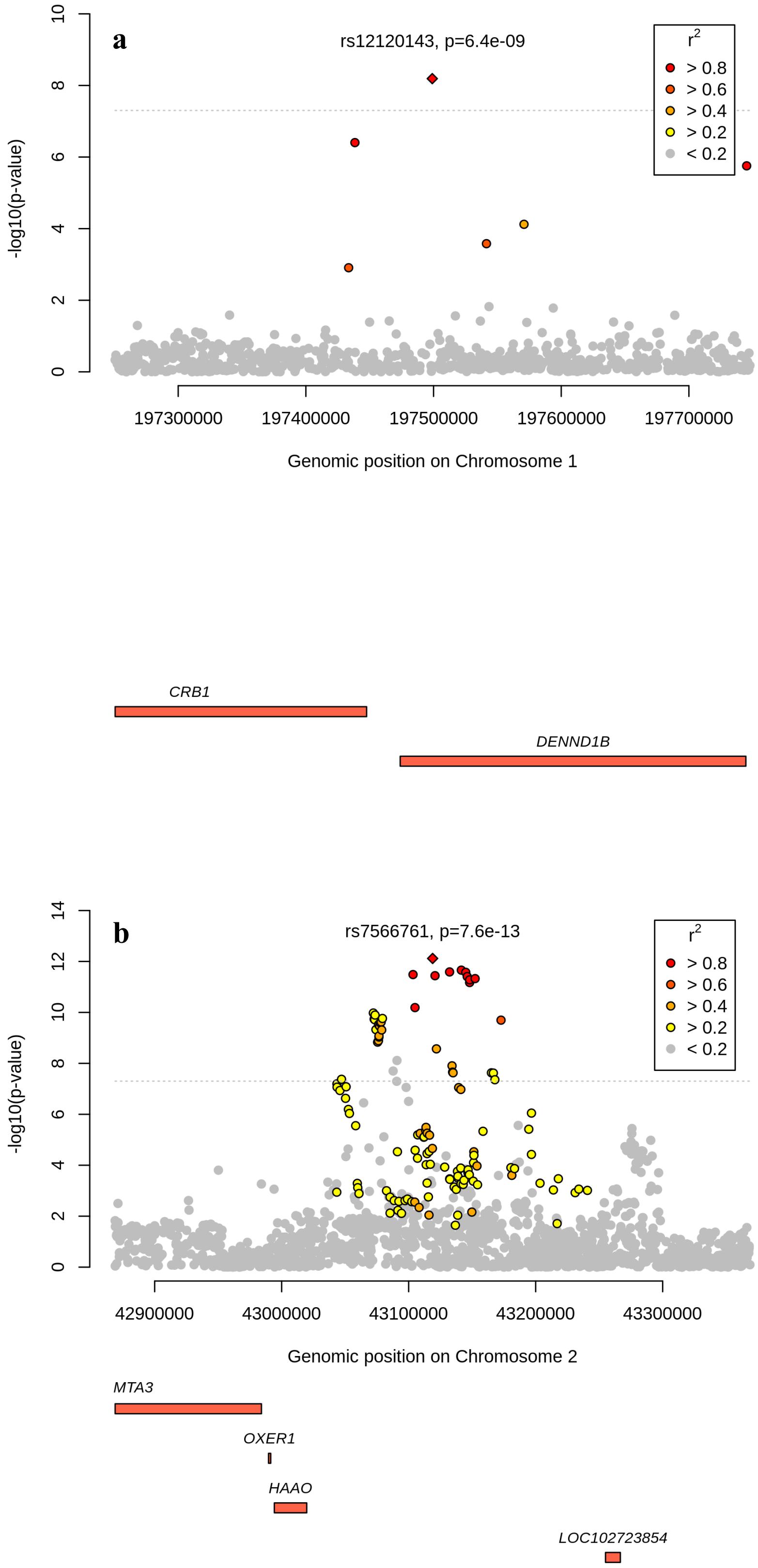
a
rs12120143, p=6.4e-09
-log10(p-value)
r2
> 0.8
> 0.6
> 0.4
> 0.2
< 0.2
197300000
197400000
197500000
197600000
197700000
Genomic position on Chromosome 1
CRB1
DENND1B
b
rs7566761, p=7.6e-13
-log10(p-value)
r2
> 0.8
> 0.6
> 0.4
> 0.2
< 0.2
42900000
43000000
43100000
43200000
43300000
Genomic position on Chromosome 2
MTA3
OXER1
HAAO
LOC102723854

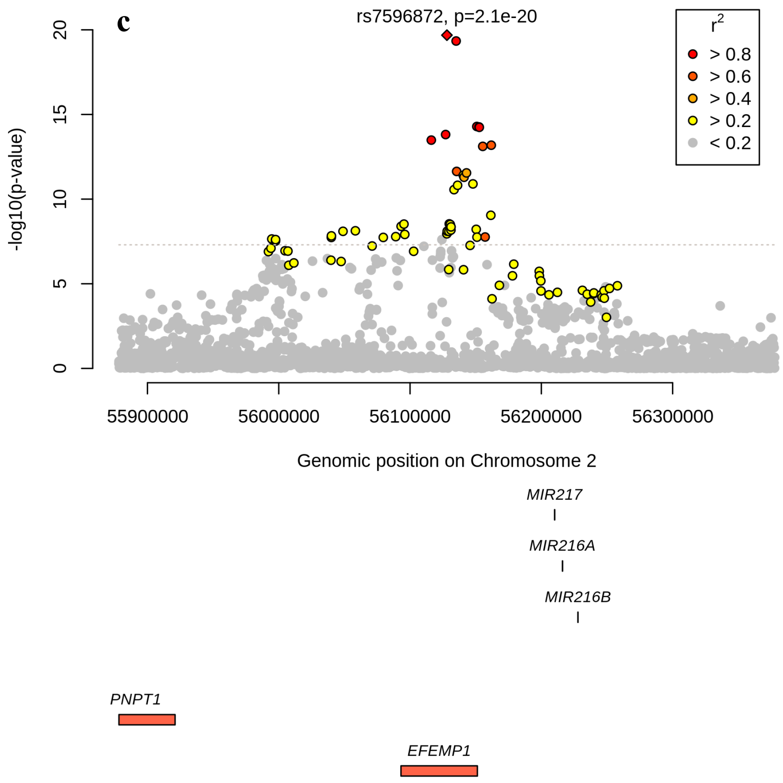
c
rs7596872, p=2.1e-20
r2
> 0.8
> 0.6
> 0.4
> 0.2
< 0.2
-log10(p-value)
0
5
10
15
20
55900000
56000000
56100000
56200000
56300000
Genomic position on Chromosome 2
MIR217
MIR216A
MIR216B
PNPT1
EFEMP1

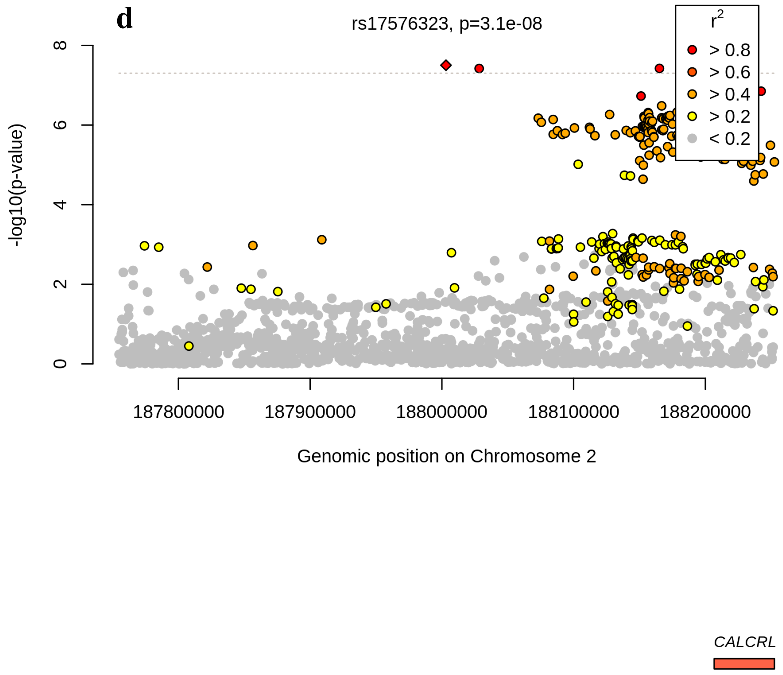
d
rs17576323, p=3.1e-08
r2
> 0.8
> 0.6
> 0.4
> 0.2
< 0.2
-log10(p-value)
0
2
4
6
8
187800000
187900000
188000000
188100000
188200000
Genomic position on Chromosome 2
CALCRL

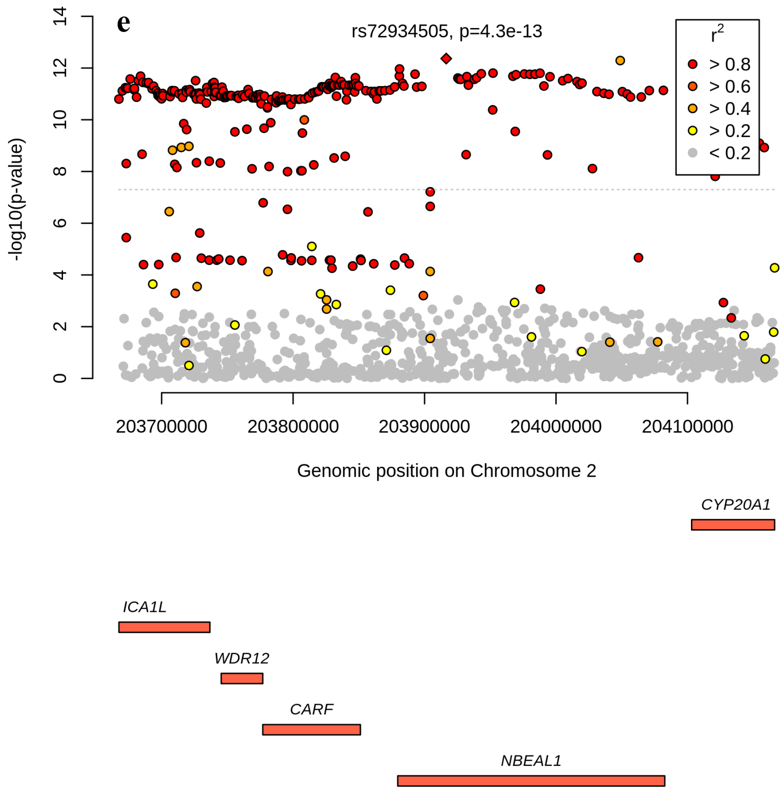
e
rs72934505, p=4.3e-13
-log10(p-value)
0
2
4
6
8
10
12
14
r2
> 0.8
> 0.6
> 0.4
> 0.2
< 0.2
203700000
203800000
203900000
204000000
204100000
Genomic position on Chromosome 2
CYP20A1
ICA1L
WDR12
CARF
NBEAL1

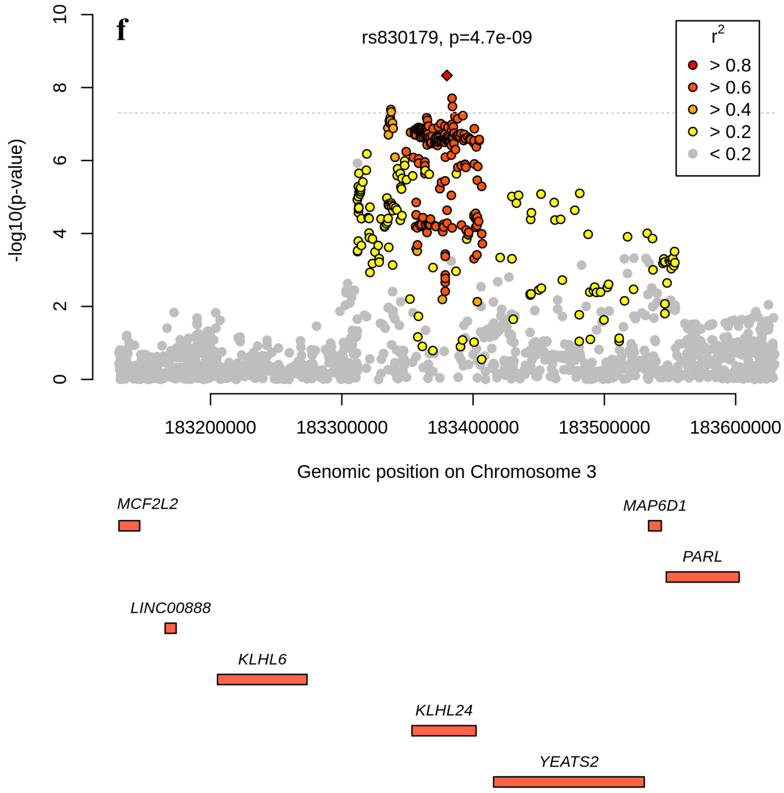
f
rs830179, p=4.7e-09
-log10(p-value)
0
2
4
6
8
10
r2
> 0.8
> 0.6
> 0.4
> 0.2
< 0.2
183200000
183300000
183400000
183500000
183600000
Genomic position on Chromosome 3
MCF2L2
MAP6D1
PARL
LINC00888
KLHL6
KLHL24
YEATS2

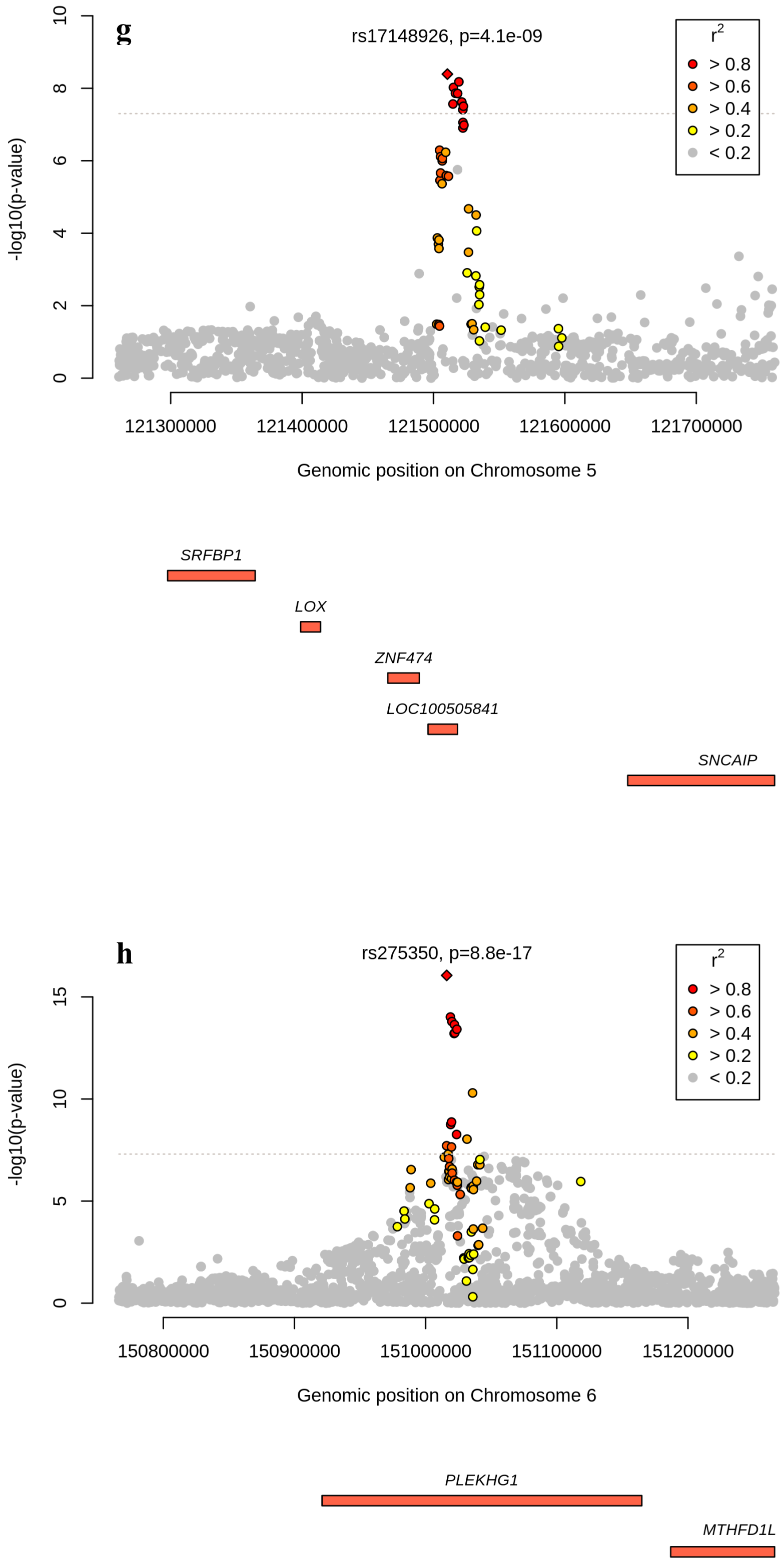
g
rs17148926, p=4.1e-09
-log10(p-value)
r2
> 0.8
> 0.6
> 0.4
> 0.2
< 0.2
121300000
121400000
121500000
121600000
121700000
Genomic position on Chromosome 5
SRFBP1
LOX
ZNF474
LOC100505841
SNCAIP
h
rs275350, p=8.8e-17
-log10(p-value)
r2
> 0.8
> 0.6
> 0.4
> 0.2
< 0.2
150800000
150900000
151000000
151100000
151200000
Genomic position on Chromosome 6
PLEKHG1
MTHFD1L

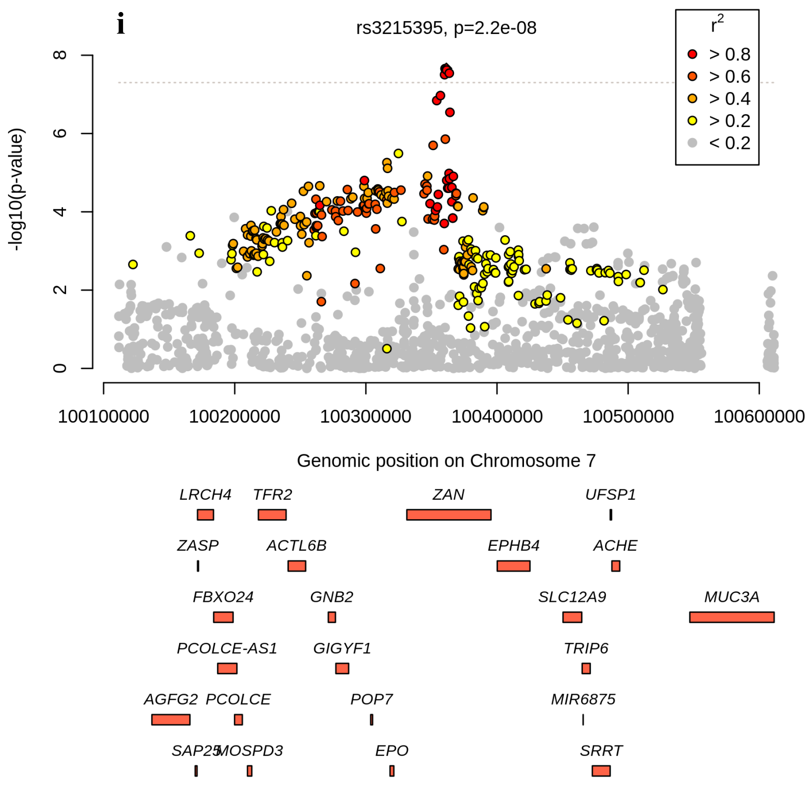
i
rs3215395, p=2.2e-08
r2
> 0.8
> 0.6
> 0.4
> 0.2
< 0.2
-log10(p-value)
0
2
4
6
8
100100000
100200000
100300000
100400000
100500000
100600000
Genomic position on Chromosome 7
LRCH4
TFR2
ZAN
UFSP1
ZASP
ACTL6B
EPHB4
ACHE
FBXO24
GNB2
SLC12A9
MUC3A
PCOLCE-AS1
GIGYF1
TRIP6
AGFG2
PCOLCE
POP7
MIR6875
SAP25
MOSPD3
EPO
SRRT

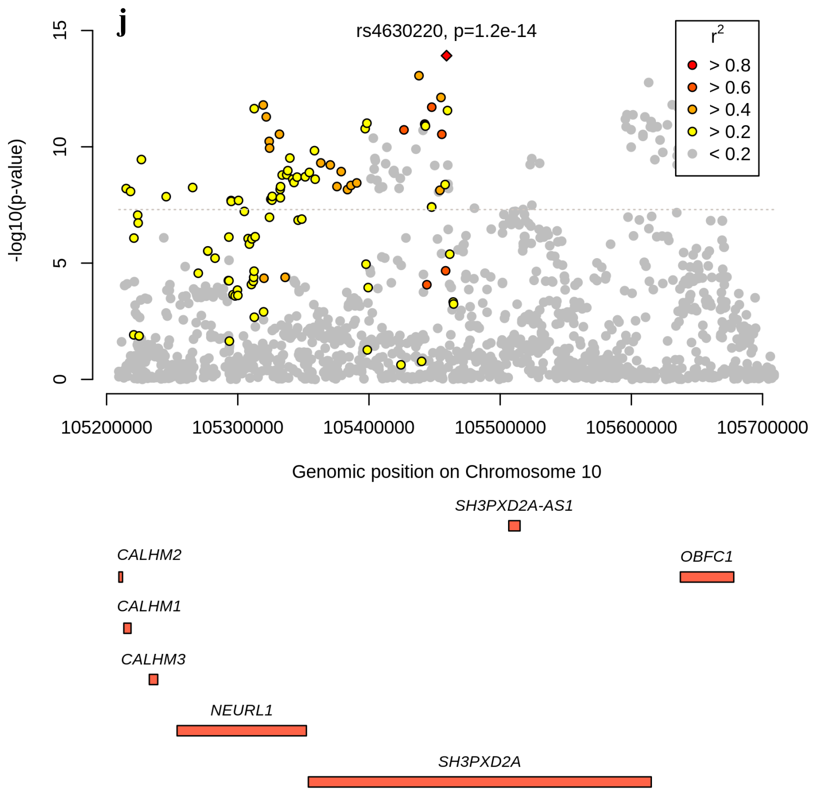
j
rs4630220, p=1.2e-14
r2
> 0.8
> 0.6
> 0.4
> 0.2
< 0.2
-log10(p-value)
0
5
10
15
105200000
105300000
105400000
105500000
105600000
105700000
Genomic position on Chromosome 10
SH3PXD2A-AS1
CALHM2
OBFC1
CALHM1
CALHM3
NEURL1
SH3PXD2A

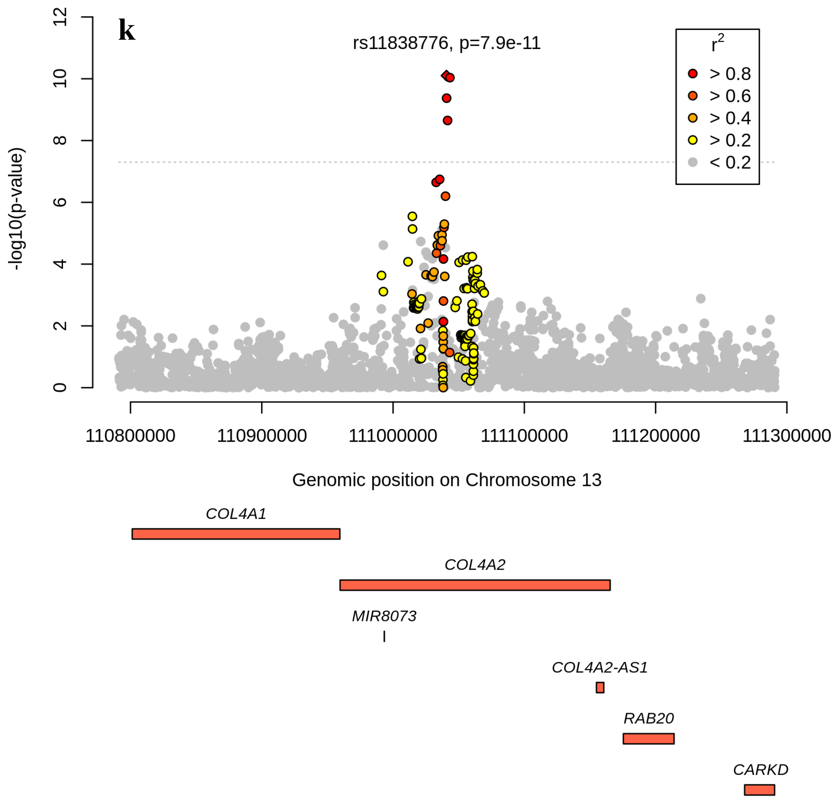
k
rs11838776, p=7.9e-11
r2
> 0.8
> 0.6
> 0.4
> 0.2
< 0.2
-log10(p-value)
0
2
4
6
8
10
12
110800000
110900000
111000000
111100000
111200000
111300000
Genomic position on Chromosome 13
COL4A1
COL4A2
MIR8073
COL4A2-AS1
RAB20
CARKD

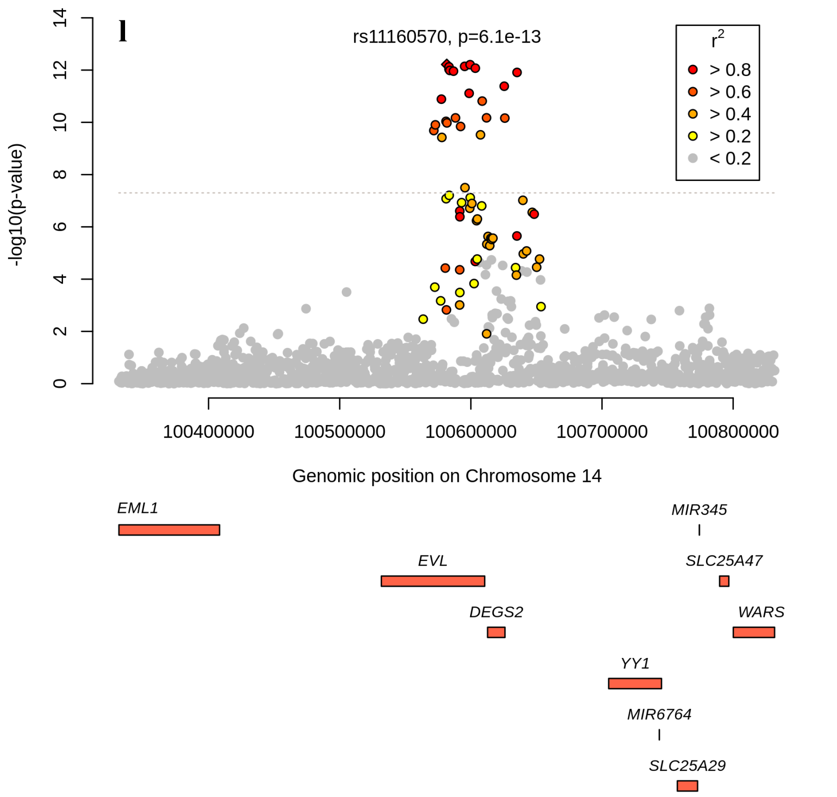
l
rs11160570, p=6.1e-13
r2
> 0.8
> 0.6
> 0.4
> 0.2
< 0.2
-log10(p-value)
0
2
4
6
8
10
12
14
100400000
100500000
100600000
100700000
100800000
Genomic position on Chromosome 14
EML1
MIR345
EVL
SLC25A47
DEGS2
WARS
YY1
MIR6764
SLC25A29

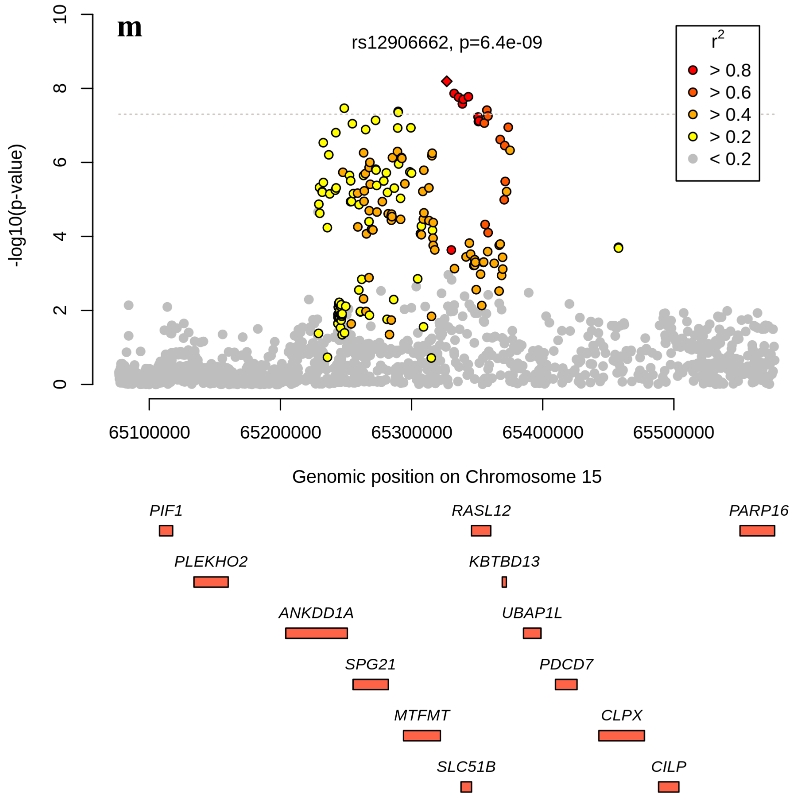
m
rs12906662, p=6.4e-09
-log10(p-value)
r2
> 0.8
> 0.6
> 0.4
> 0.2
< 0.2
0
2
4
6
8
10
65100000
65200000
65300000
65400000
65500000
Genomic position on Chromosome 15
PIF1
RASL12
PARP16
PLEKHO2
KBTBD13
ANKDD1A
UBAP1L
SPG21
PDCD7
MTFMT
CLPX
SLC51B
CILP

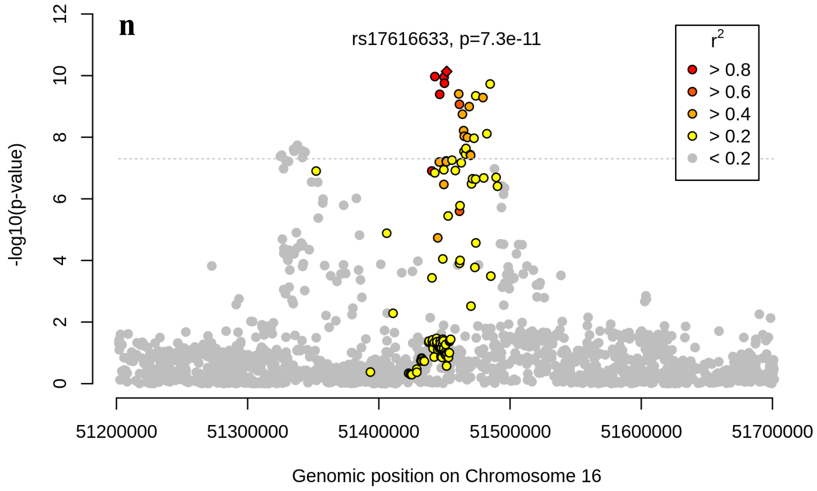
n
rs17616633, p=7.3e-11
-log10(p-value)
r2
> 0.8
> 0.6
> 0.4
> 0.2
< 0.2
0
2
4
6
8
10
12
51200000
51300000
51400000
51500000
51600000
51700000
Genomic position on Chromosome 16

o

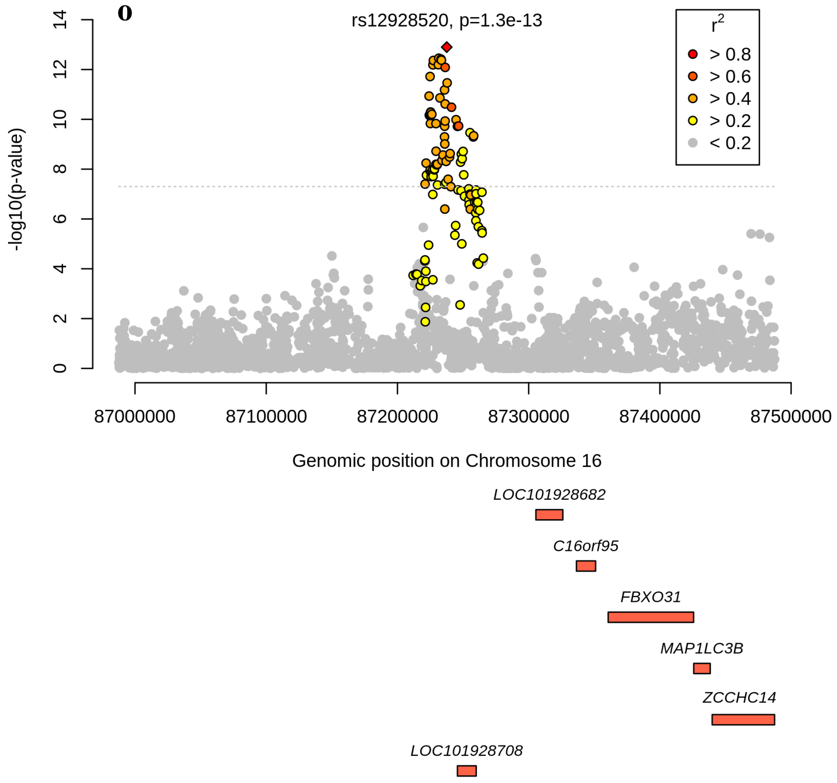

rs12928520, p=1.3e-13
r2
> 0.8
> 0.6
> 0.4
> 0.2
< 0.2
-log10(p-value)
0
2
4
6
8
10
12
14
87000000
87100000
87200000
87300000
87400000
87500000
Genomic position on Chromosome 16
LOC101928682
C16orf95
FBXO31
MAP1LC3B
ZCCHC14
LOC101928708

p

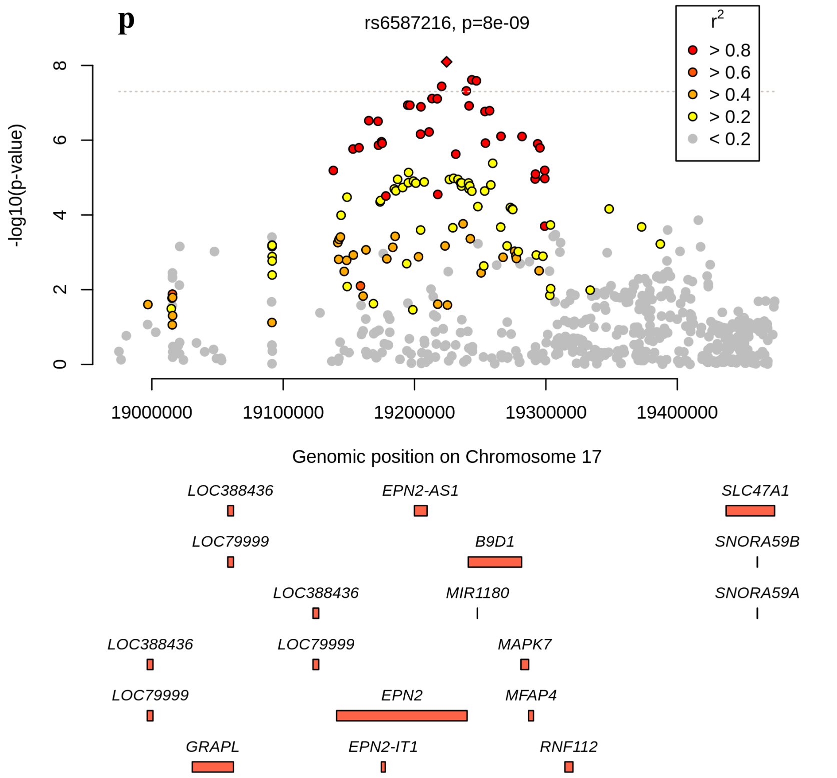

rs6587216, p=8e-09
r2
> 0.8
> 0.6
> 0.4
> 0.2
< 0.2
-log10(p-value)
0
2
4
6
8
19000000
19100000
19200000
19300000
19400000
Genomic position on Chromosome 17
LOC388436
EPN2-AS1
SLC47A1
LOC79999
B9D1
SNORA59B
LOC388436
MIR1180
SNORA59A
LOC388436
LOC79999
MAPK7
LOC79999
EPN2
MFAP4
GRAPL
EPN2-IT1
RNF112

q

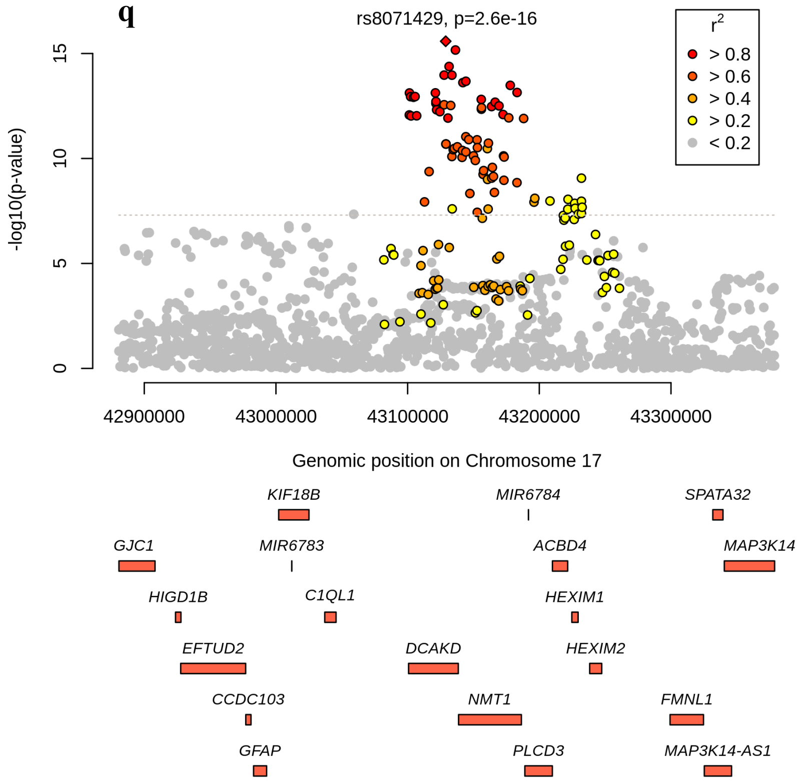
rs8071429, p=2.6e-16
r2
> 0.8
> 0.6
> 0.4
> 0.2
< 0.2
-log10(p-value)
Genomic position on Chromosome 17
KIF18B
MIR6784
SPATA32
GJC1
MIR6783
ACBD4
MAP3K14
HIGD1B
C1QL1
HEXIM1
EFTUD2
DCAKD
HEXIM2
CCDC103
NMT1
FMNL1
GFAP
PLCD3
MAP3K14-AS1

r

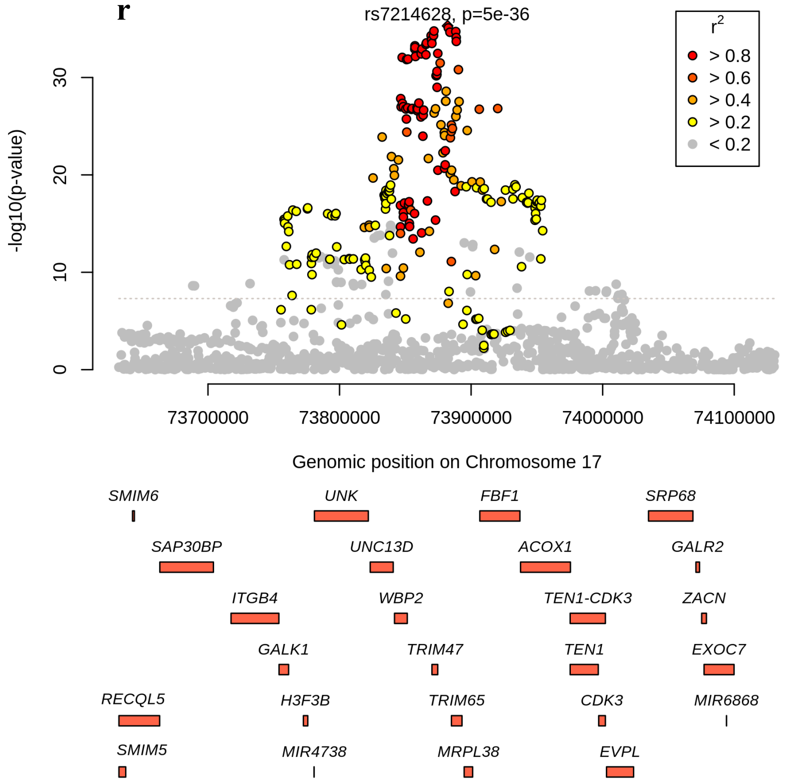
rs7214628, p=5e-36
r2
> 0.8
> 0.6
> 0.4
> 0.2
< 0.2
-log10(p-value)
Genomic position on Chromosome 17
SMIM6
UNK
FBF1
SRP68
SAP30BP
UNC13D
ACOX1
GALR2
ITGB4
WBP2
TEN1-CDK3
ZACN
GALK1
TRIM47
TEN1
EXOC7
RECQL5
H3F3B
TRIM65
CDK3
MIR6868
SMIM5
MIR4738
MRPL38
EVPL

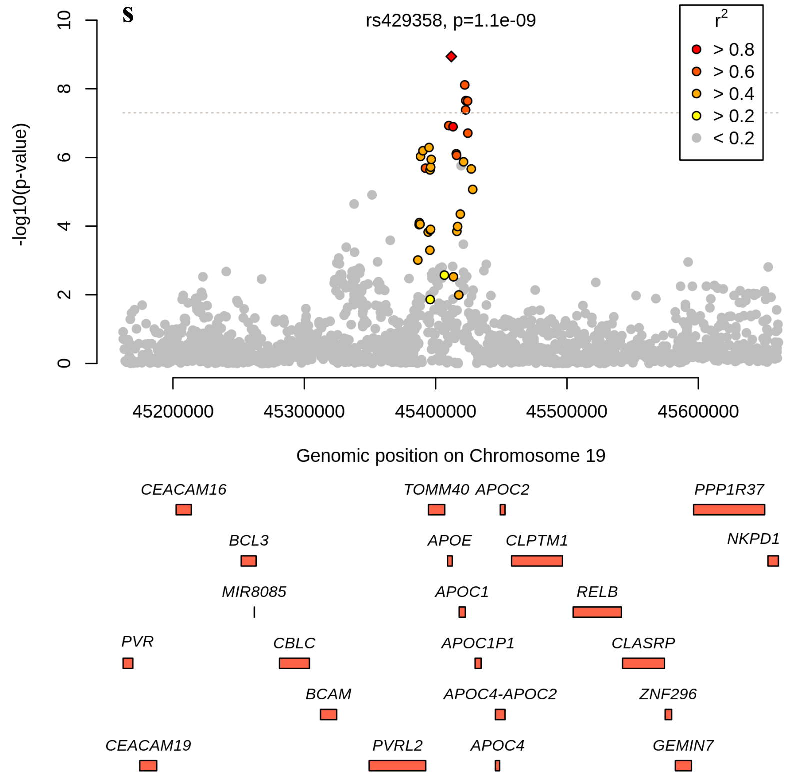

Supplementary Figure 2. Regional plots of WMH significant association results. The different panels correspond to the following top SNPs: **a.** rs12120143, **b.** rs7566761, **c.** rs7596872, **d.** rs17576323, **e.** rs72934505, **f.** rs830179, **g.** rs17148926, **h.** rs275350, **i.** rs3215395, **j.** rs4630220, **k.** rs11838776, **l.** rs11160570, **m.** rs12906662, **n.** rs17616633, **o.** rs12928520, **p.** rs6587216, **q.** rs8071429, **r.** rs7214628 and **s.** rs429358.

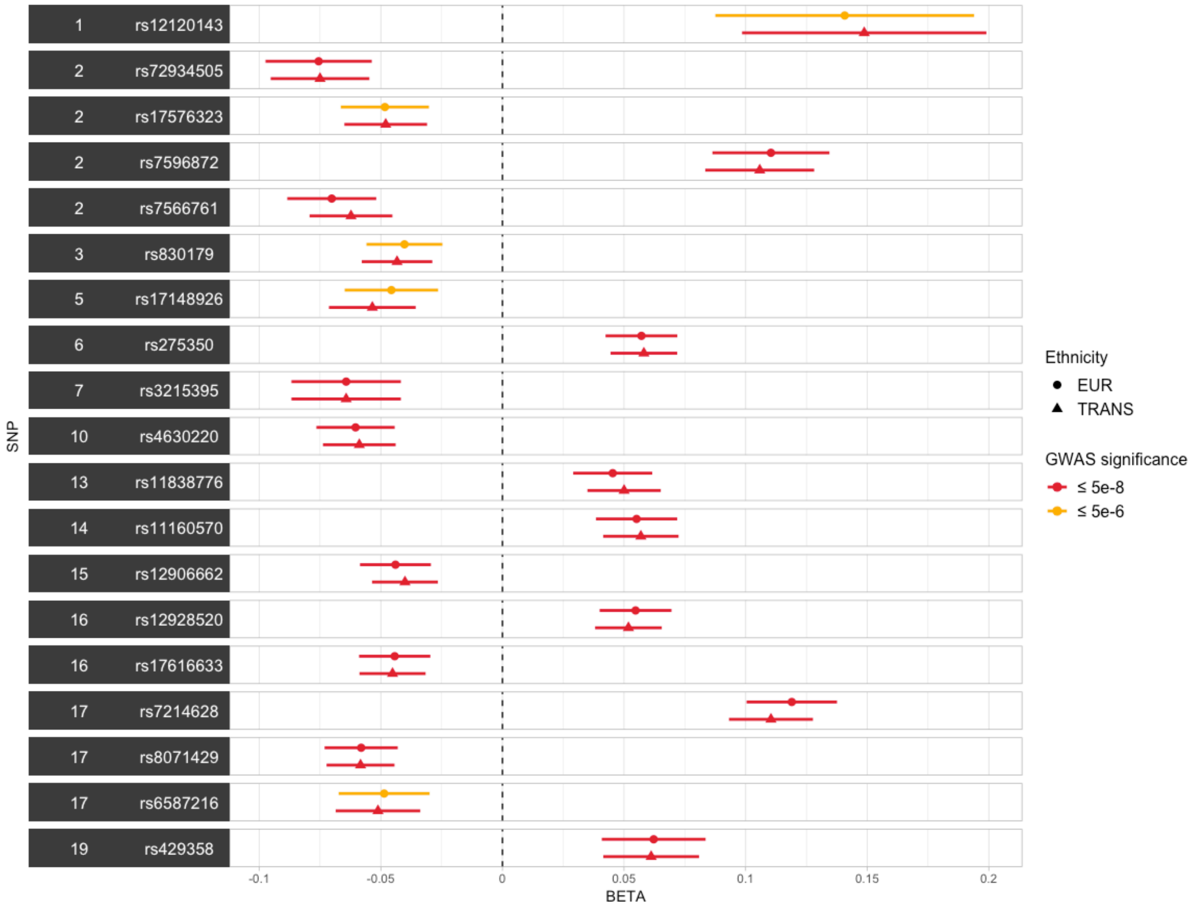

Supplementary Figure 3. Comparison between European and trans-ethnic results for WMH reported top SNPs. Beta coefficients were estimated from meta-analysis Z-scores and sample sizes, and allele frequencies in UK Biobank. Error bars, mean ± 1.96 s.e..

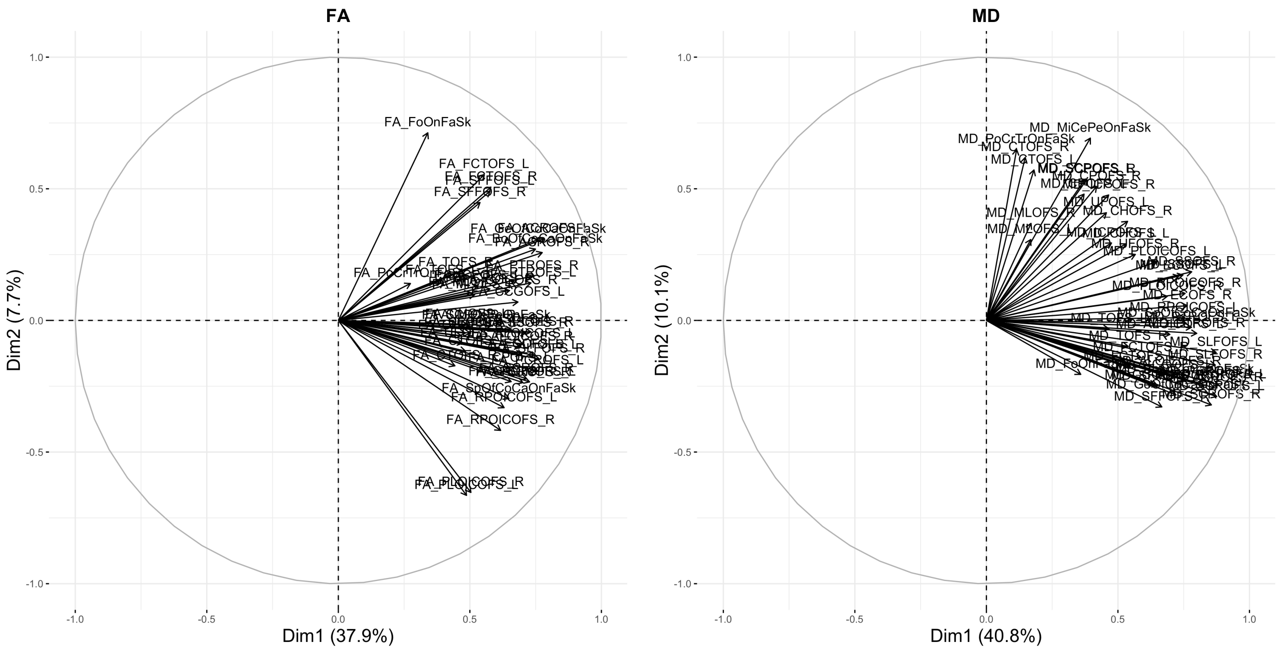

Supplementary Figure 4. PCA correlation plots.

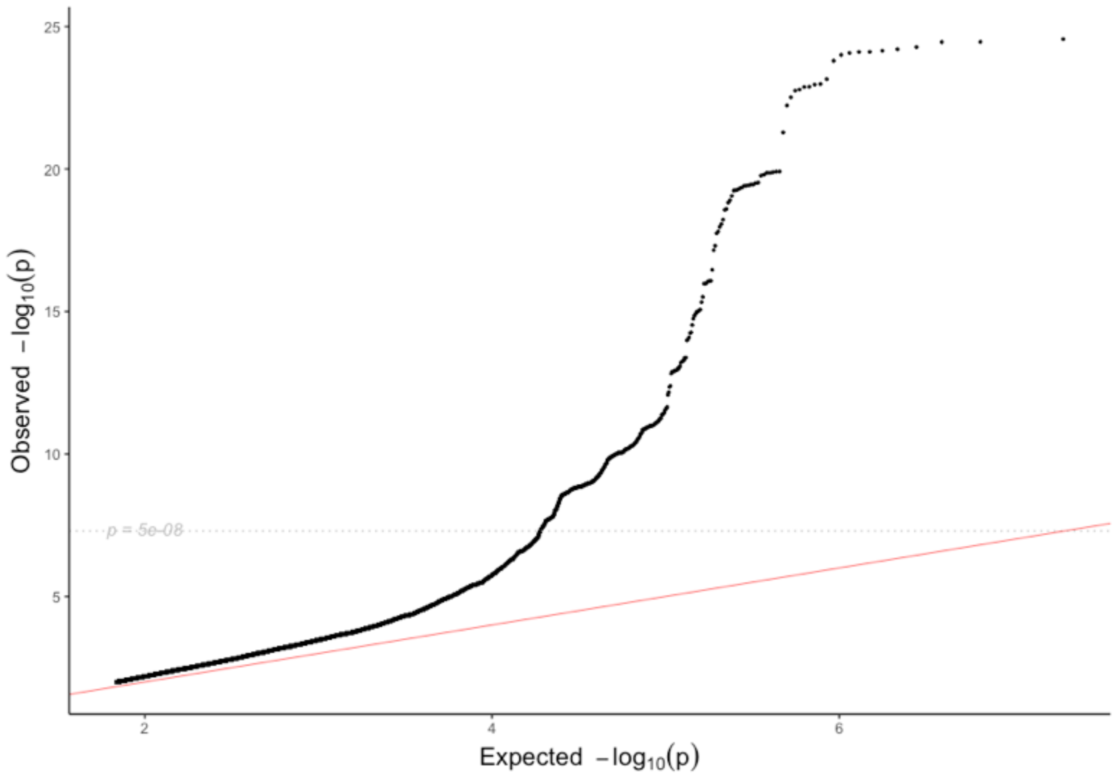

Supplementary Figure 5. QQ plot for FA GWAS in UK Biobank.

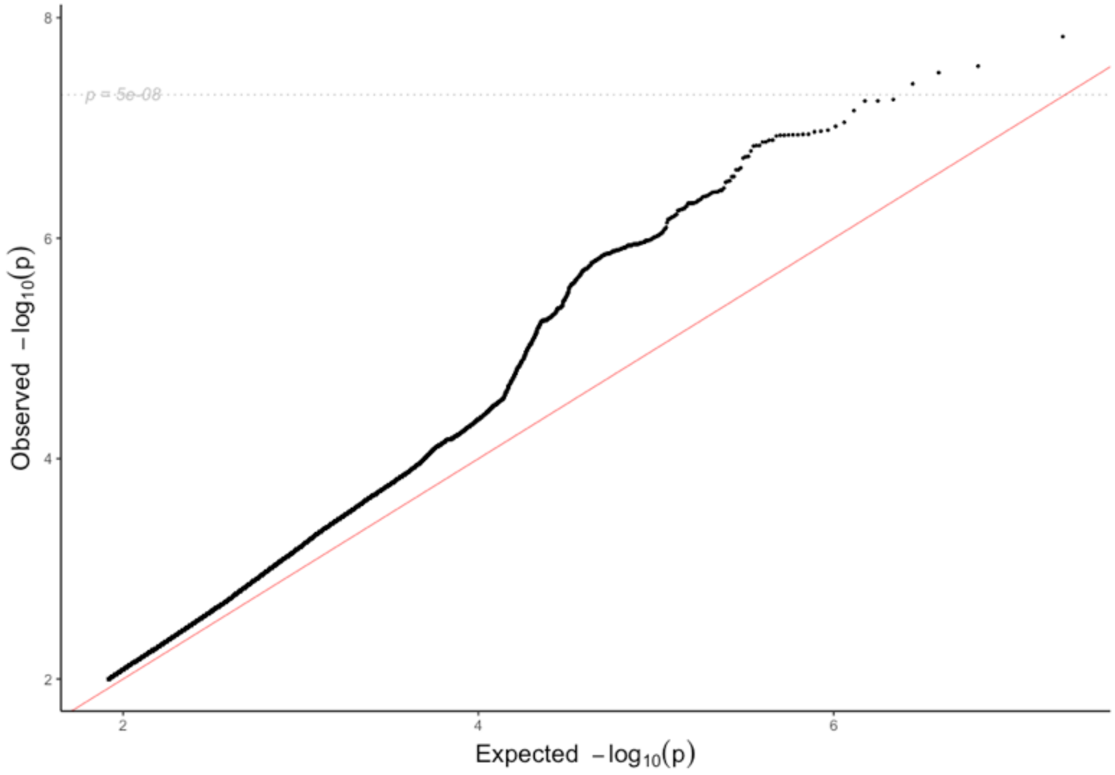

Supplementary Figure 6. QQ plot for MD GWAS in UK Biobank.

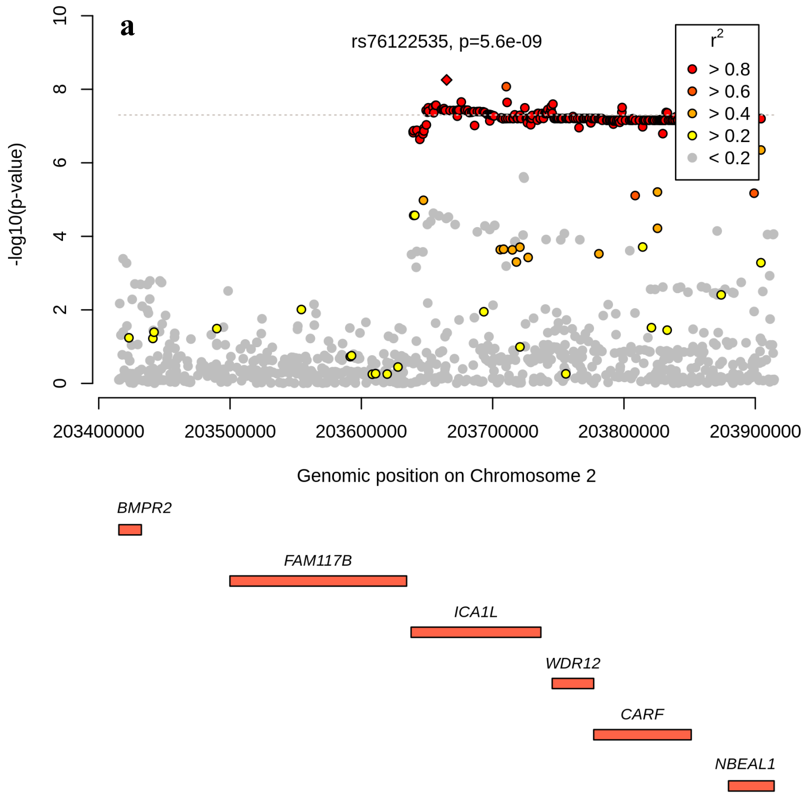

a
rs76122535, p=5.6e-09
r2
> 0.8
> 0.6
> 0.4
> 0.2
< 0.2
-log10(p-value)
0
2
4
6
8
10
203400000
203500000
203600000
203700000
203800000
203900000
Genomic position on Chromosome 2
BMPR2
FAM117B
ICA1L
WDR12
CARF
NBEAL1

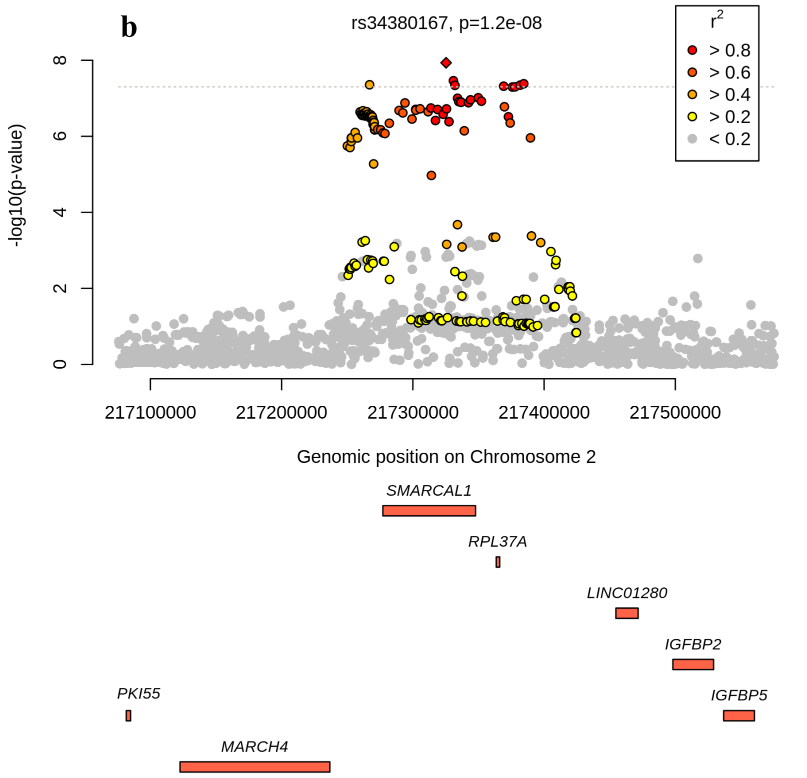

b
rs34380167, p=1.2e-08
r2
> 0.8
> 0.6
> 0.4
> 0.2
< 0.2
-log10(p-value)
0
2
4
6
8
217100000
217200000
217300000
217400000
217500000
Genomic position on Chromosome 2
SMARCAL1
RPL37A
LINC01280
IGFBP2
PKI55
IGFBP5
MARCH4

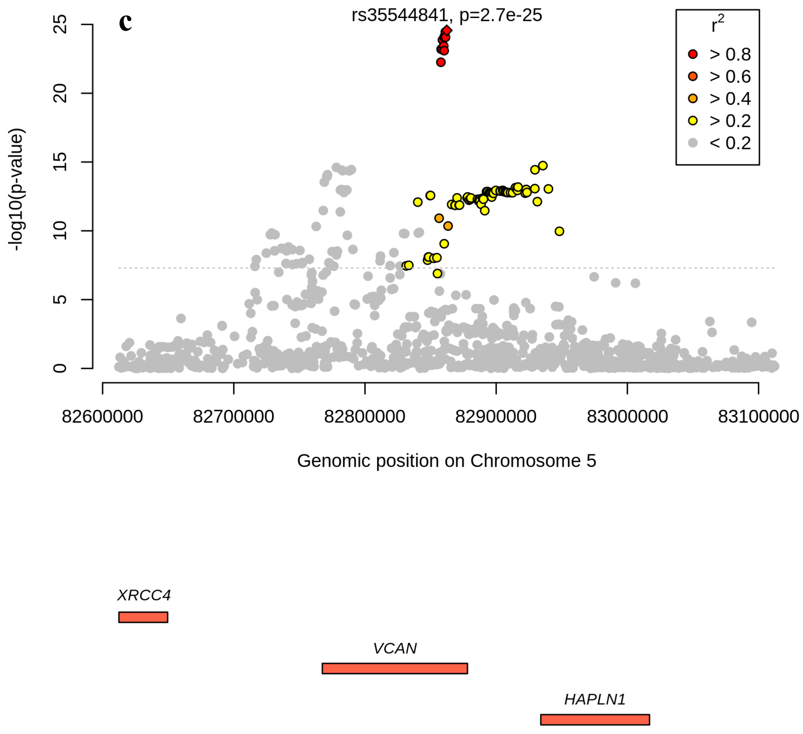
c
rs35544841, p=2.7e-25
r2
> 0.8
> 0.6
> 0.4
> 0.2
< 0.2
-log10(p-value)
0
5
10
15
20
25
82600000
82700000
82800000
82900000
83000000
83100000
Genomic position on Chromosome 5
XRCC4
VCAN
HAPLN1

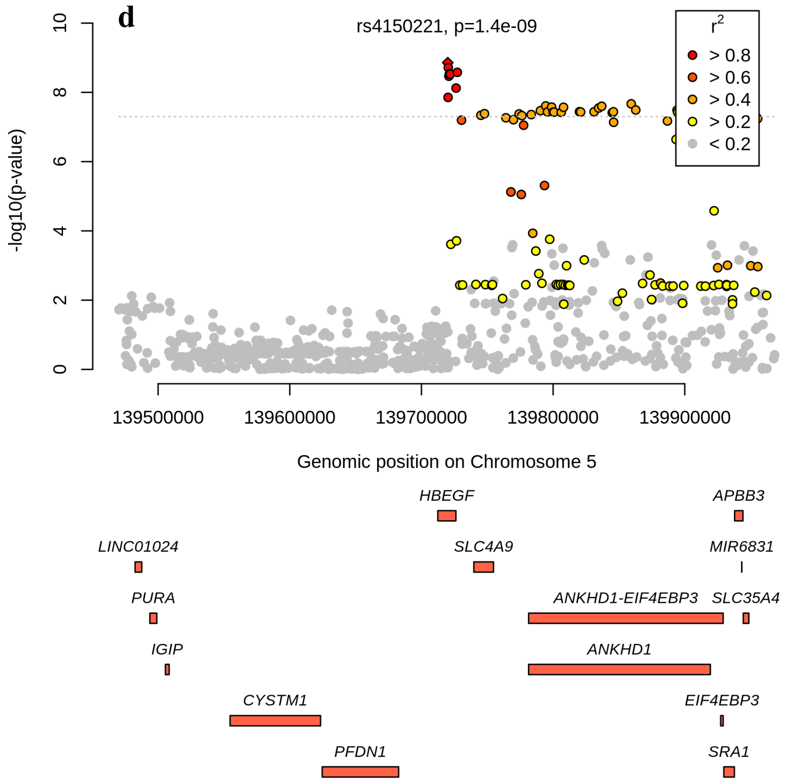
d
rs4150221, p=1.4e-09
r2
> 0.8
> 0.6
> 0.4
> 0.2
< 0.2
-log10(p-value)
0
2
4
6
8
10
139500000
139600000
139700000
139800000
139900000
Genomic position on Chromosome 5
HBEGF
APBB3
LINC01024
SLC4A9
MIR6831
PURA
ANKHD1-EIF4EBP3
SLC35A4
IGIP
ANKHD1
CYSTM1
EIF4EBP3
PFDN1
SRA1

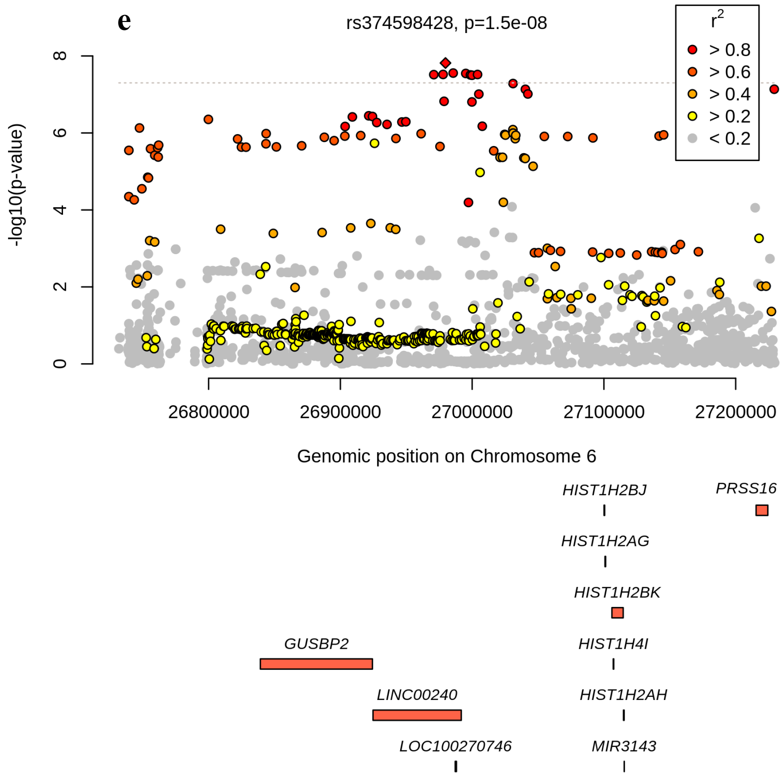
e
rs374598428, p=1.5e-08
r2
> 0.8
> 0.6
> 0.4
> 0.2
< 0.2
-log10(p-value)
0
2
4
6
8
26800000
26900000
27000000
27100000
27200000
Genomic position on Chromosome 6
HIST1H2BJ
PRSS16
HIST1H2AG
HIST1H2BK
GUSBP2
HIST1H4I
LINC00240
HIST1H2AH
LOC100270746
MIR3143

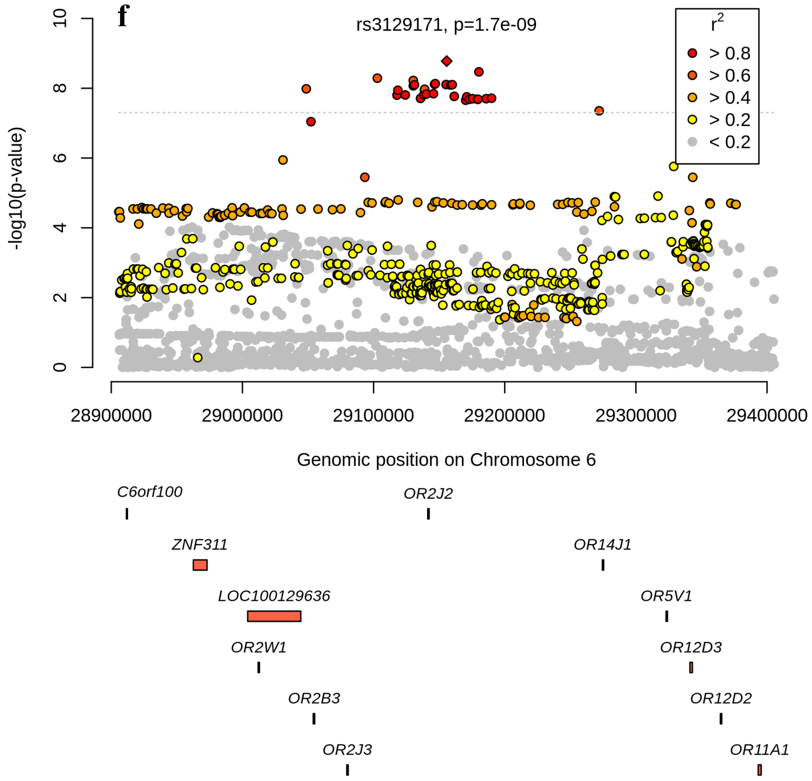
f
rs3129171, p=1.7e-09
r2
> 0.8
> 0.6
> 0.4
> 0.2
< 0.2
-log10(p-value)
0
2
4
6
8
10
28900000
29000000
29100000
29200000
29300000
29400000
Genomic position on Chromosome 6
C6orf100
OR2J2
ZNF311
OR14J1
LOC100129636
OR5V1
OR2W1
OR12D3
OR2B3
OR12D2
OR2J3
OR11A1

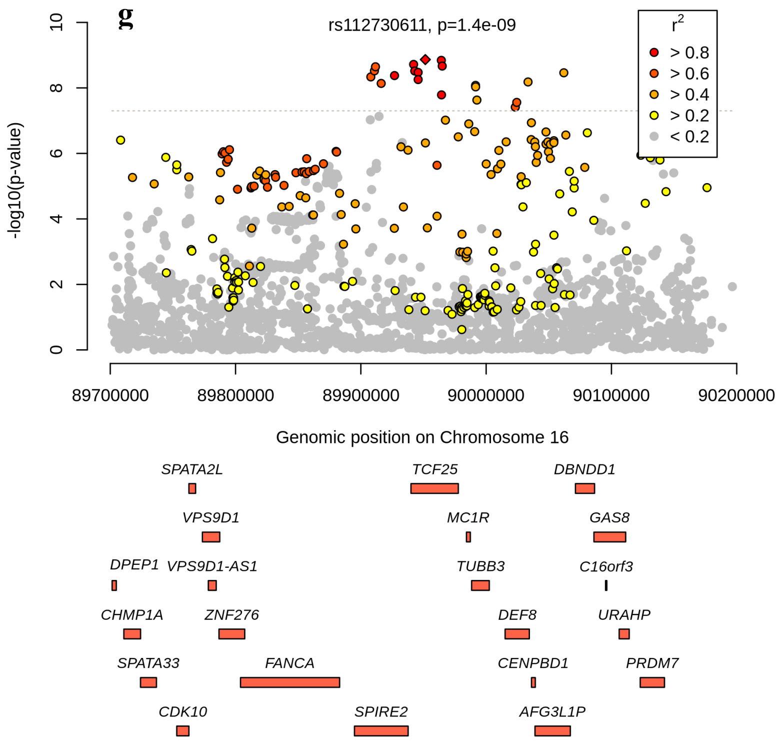
g
rs112730611, p=1.4e-09
-log10(p-value)
r2
> 0.8
> 0.6
> 0.4
> 0.2
< 0.2
89700000
89800000
89900000
90000000
90100000
90200000
Genomic position on Chromosome 16
SPATA2L
TCF25
DBNDD1
VPS9D1
MC1R
GAS8
DPEP1
VPS9D1-AS1
TUBB3
C16orf3
CHMP1A
ZNF276
DEF8
URAHP
SPATA33
FANCA
CENPBD1
PRDM7
CDK10
SPIRE2
AFG3L1P

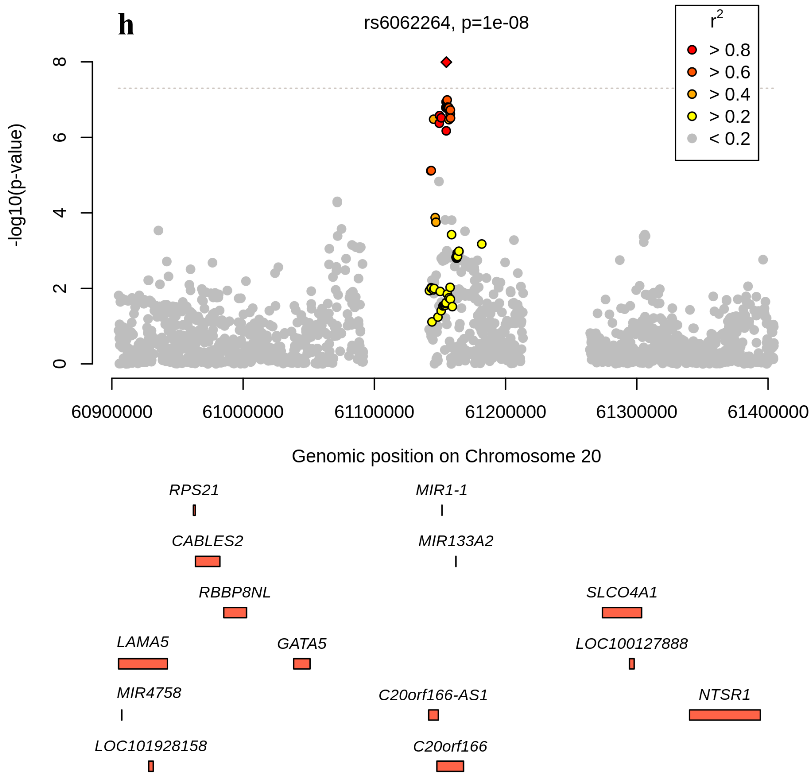
h
rs6062264, p=1e-08
-log10(p-value)
r2
> 0.8
> 0.6
> 0.4
> 0.2
< 0.2
60900000
61000000
61100000
61200000
61300000
61400000
Genomic position on Chromosome 20
RPS21
MIR1-1
CABLES2
MIR133A2
RBBP8NL
SLCO4A1
LAMA5
GATA5
LOC100127888
MIR4758
C20orf166-AS1
NTSR1
LOC101928158
C20orf166

Supplementary Figure 7. Regional plots of FA significant association results. The different panels correspond to the following top SNPs: **a.** rs76122535, **b.** rs34380167, **c.** rs35544841, **d.** rs4150221, **e.** rs374598428, **f.** rs3129171, **g.** rs112730611 and **h.** rs6062264.

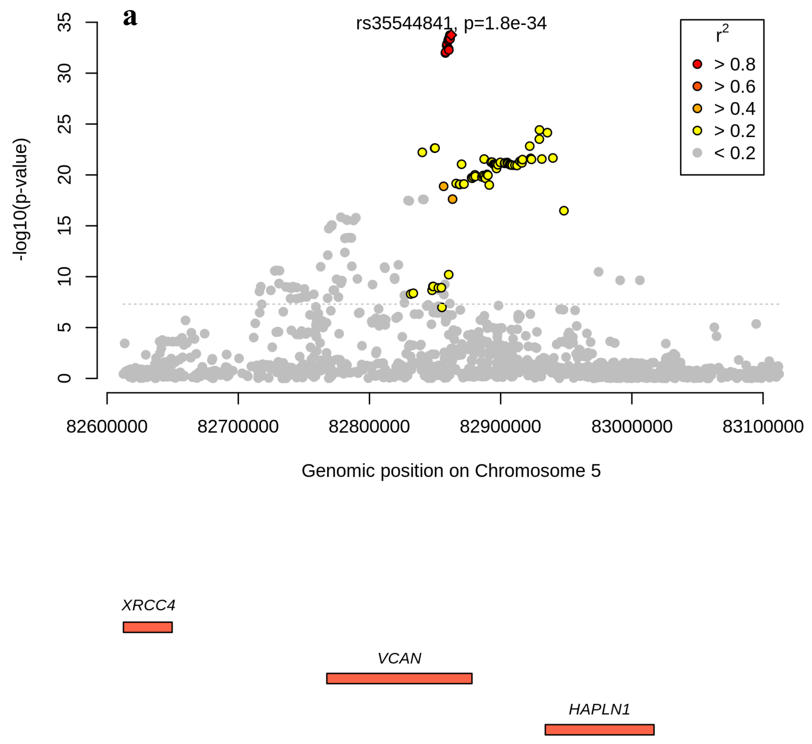
a
rs35544841, p=1.8e-34
r2
> 0.8
> 0.6
> 0.4
> 0.2
< 0.2
-log10(p-value)
Genomic position on Chromosome 5
82600000
82700000
82800000
82900000
83000000
83100000
XRCC4
VCAN
HAPLN1

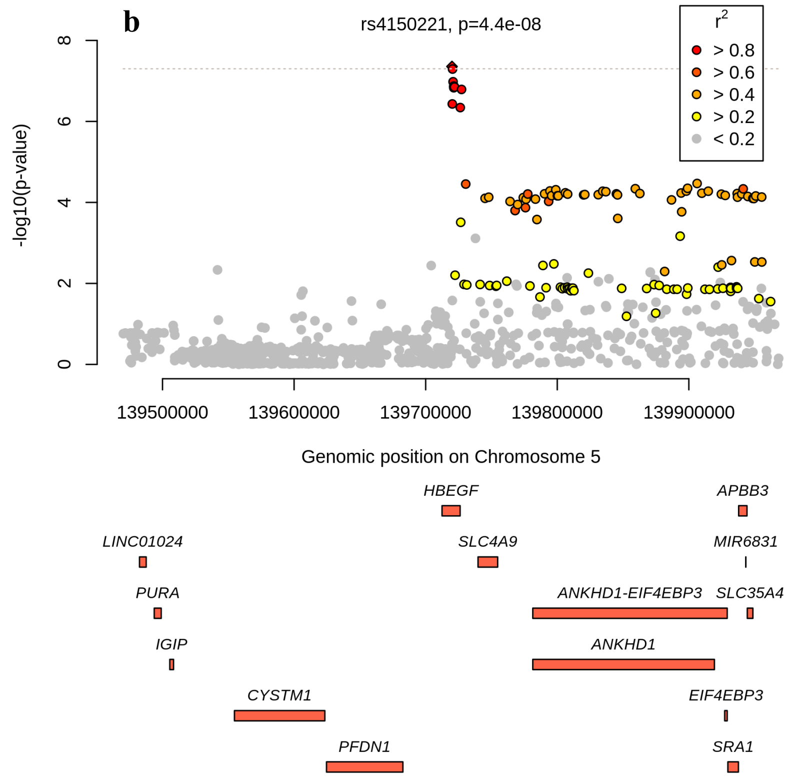
b
rs4150221, p=4.4e-08
r2
> 0.8
> 0.6
> 0.4
> 0.2
< 0.2
-log10(p-value)
Genomic position on Chromosome 5
139500000
139600000
139700000
139800000
139900000
HBEGF
APBB3
LINC01024
SLC4A9
MIR6831
PURA
ANKHD1-EIF4EBP3
SLC35A4
IGIP
ANKHD1
CYSTM1
EIF4EBP3
PFDN1
SRA1

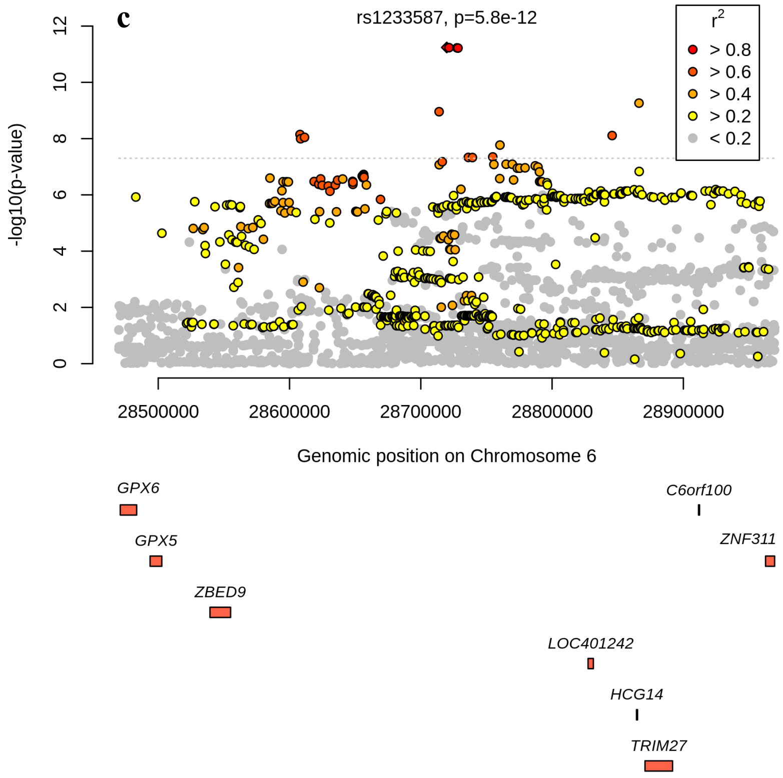
c
rs1233587, p=5.8e-12
r2
> 0.8
> 0.6
> 0.4
> 0.2
< 0.2
-log10(p-value)
0
2
4
6
8
10
12
28500000
28600000
28700000
28800000
28900000
Genomic position on Chromosome 6
GPX6
C6orf100
GPX5
ZNF311
ZBED9
LOC401242
HCG14
TRIM27

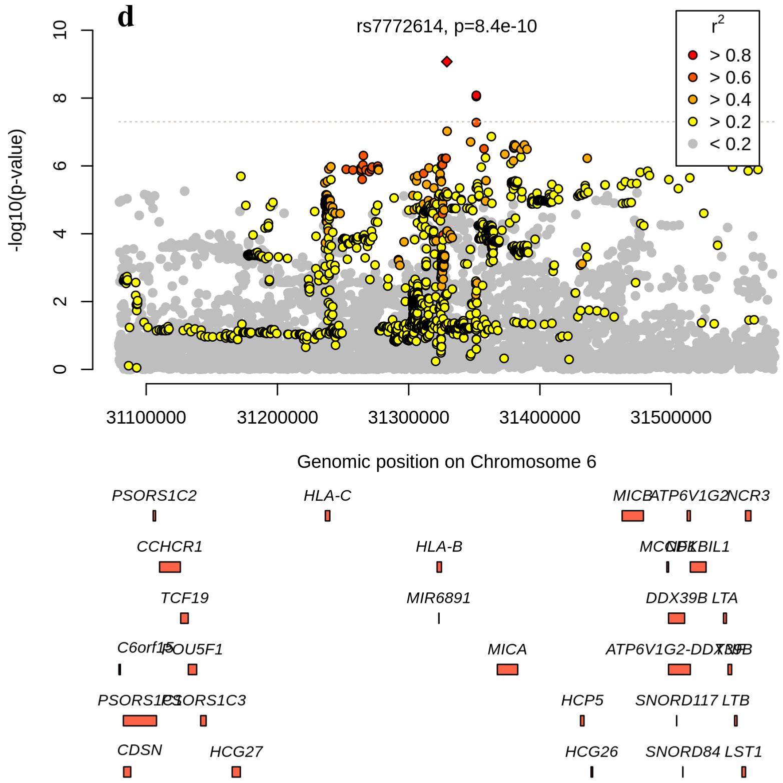
d
rs7772614, p=8.4e-10
r2
> 0.8
> 0.6
> 0.4
> 0.2
< 0.2
-log10(p-value)
0
2
4
6
8
10
31100000
31200000
31300000
31400000
31500000
Genomic position on Chromosome 6
PSORS1C2
HLA-C
MICB
ATP6V1G2
NCR3
CCHCR1
HLA-B
NFKBIL1
TCF19
MIR6891
DDX39B
LTA
C6orf15
POU5F1
MICA
ATP6V1G2-DDX39B
TNF
PSORS1C3
HCP5
SNORD117
LTB
CDSN
HCG27
HCG26
SNORD84
LST1

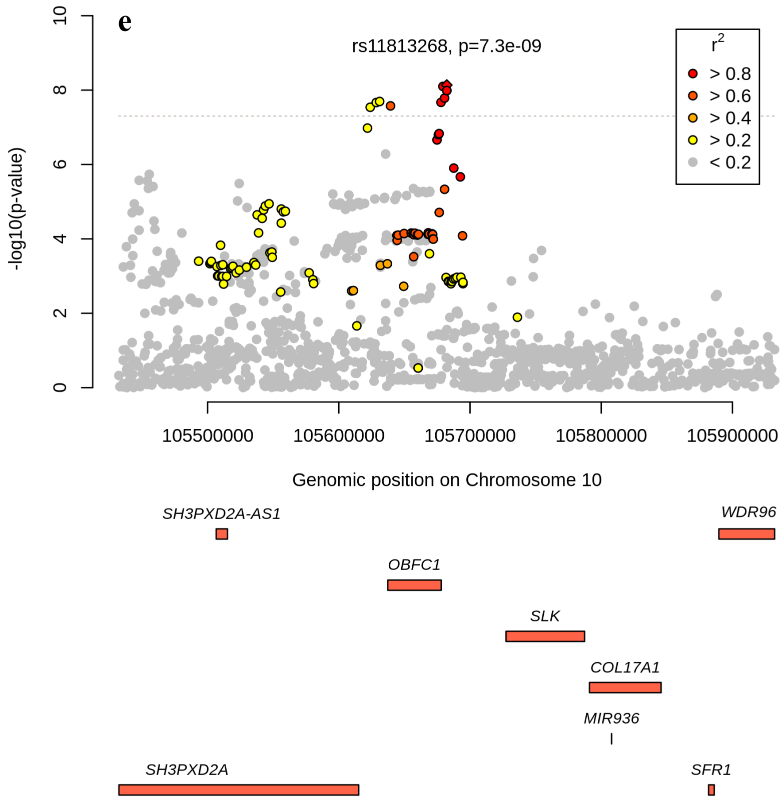
e
rs11813268, p=7.3e-09
r2
> 0.8
> 0.6
> 0.4
> 0.2
< 0.2
-log10(p-value)
0
2
4
6
8
10
105500000
105600000
105700000
105800000
105900000
Genomic position on Chromosome 10
SH3PXD2A-AS1
WDR96
OBFC1
SLK
COL17A1
MIR936
SH3PXD2A
SFR1

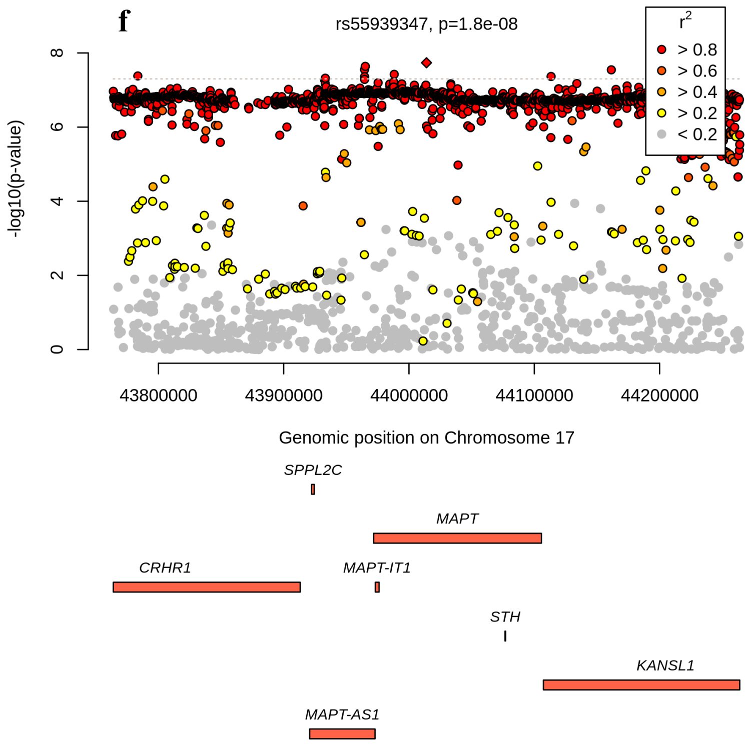
f
rs55939347, p=1.8e-08
r2
> 0.8
> 0.6
> 0.4
> 0.2
< 0.2
-log10(p-value)
0
2
4
6
8
43800000
43900000
44000000
44100000
44200000
Genomic position on Chromosome 17
SPPL2C
MAPT
CRHR1
MAPT-IT1
STH
KANSL1
MAPT-AS1

Supplementary Figure 8. Regional plots of MD significant association results. The different panels correspond to the following top SNPs: **a.** rs35544841, **b.** rs4150221, **c.** rs1233587, **d.** rs7772614, **e.** rs11813268 and **f.** rs55939347.

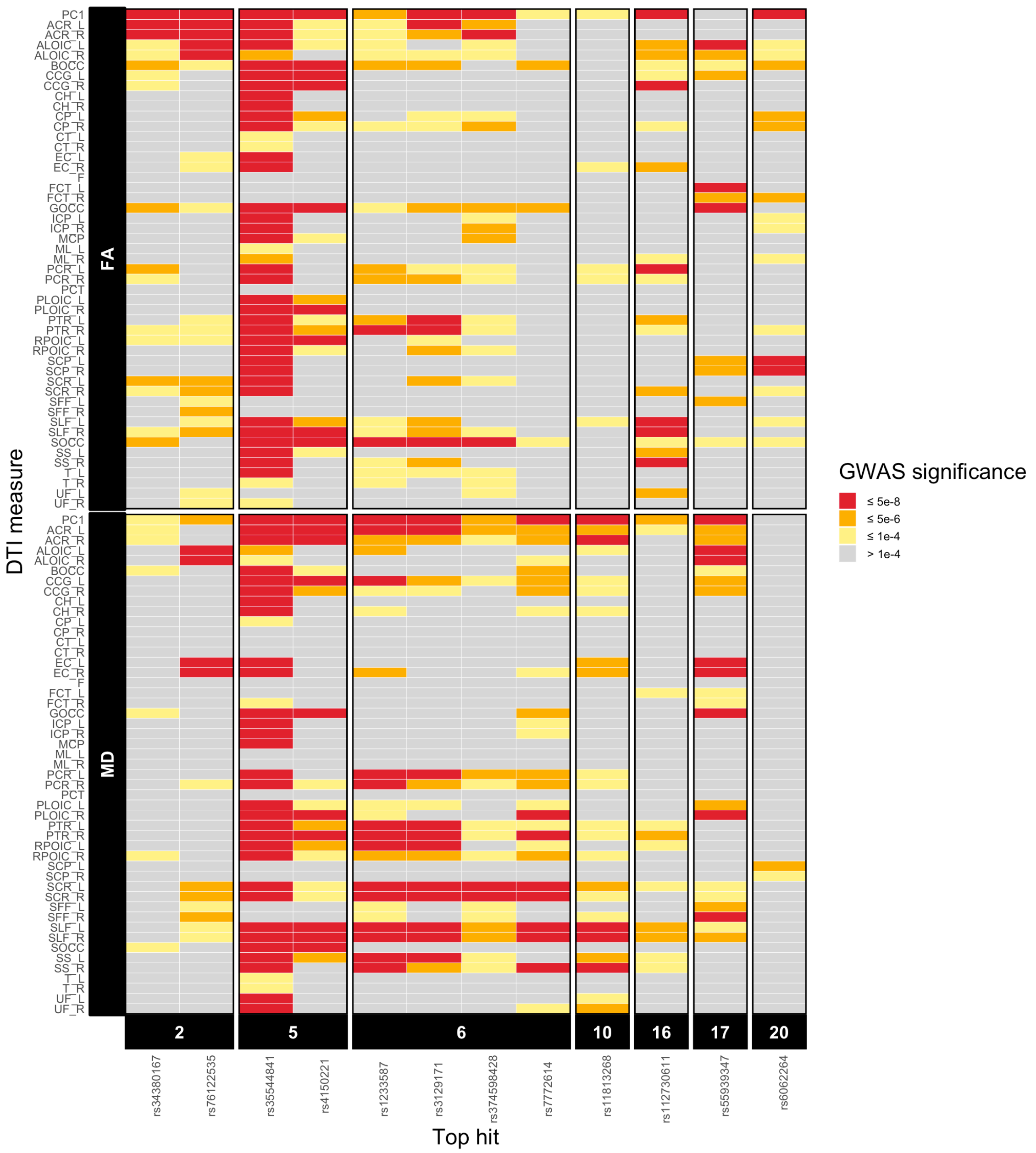

Supplementary Figure 9. Association results in the different brain regions.

Abbreviations: PC1: first principal component, as the global measure of white matter DTI signal; L: left; R: right; ACR: anterior corona radiata; ALOIC: anterior limb of internal capsule; BOCC: body of corpus callosum; CCG: cingulum cingulate gyrus; CH: cingulum hippocampus; CP: cerebral peduncle; CT: corticospinal tract; EC: external capsule; F: fornix; FCT: fornix cres/stria terminalis; GOCC: genu of corpus callosum; ICP: inferior cerebellar peduncle; MCP: middle cerebellar peduncle; ML: medial lemniscus; PCR posterior corona radiata; PCT: pontine crossing tract; PLOIC: posterior limb of internal capsule; PTR: posterior thalamic radiation; RPOIC: retrolenticular part of internal capsule; SCP: superior cerebellar peduncle; SCR: superior corona radiata; SFF: superior fronto-occipital fasciculus; SLF: superior longitudinal fasciculus; SOCC: splenium of corpus callosum; SS sagittal stratum; T: tapetum; UF: uncinate fasciculus

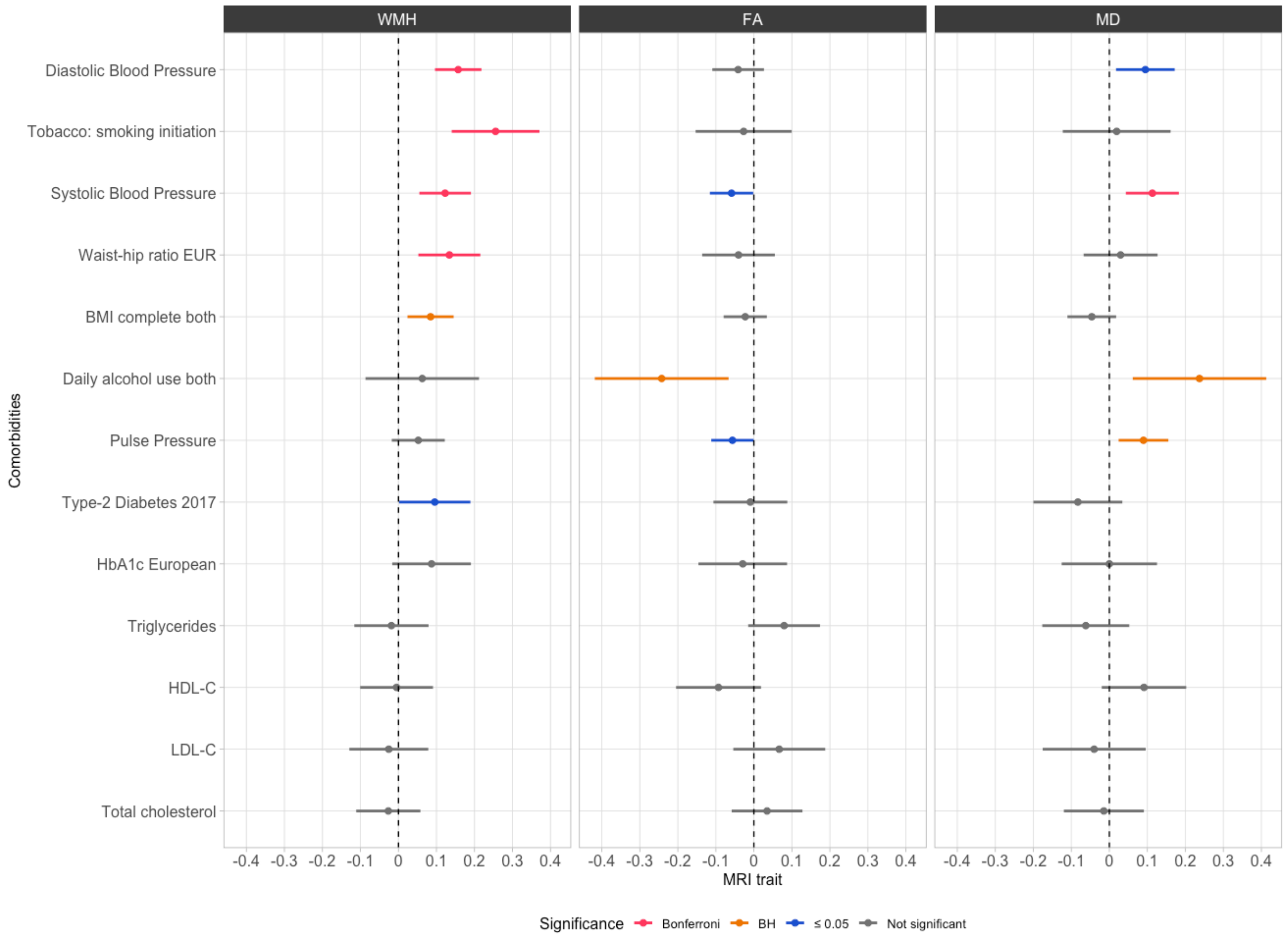

Supplementary Figure 10. Genetic correlation results with CSVD comorbidities. Bonferroni and Benjamini and Hochberg multiple testing corrections were applied considering only p-values for genetic correlation with these cardiovascular risk factors. Error bars, mean ± 1.96 s.e..

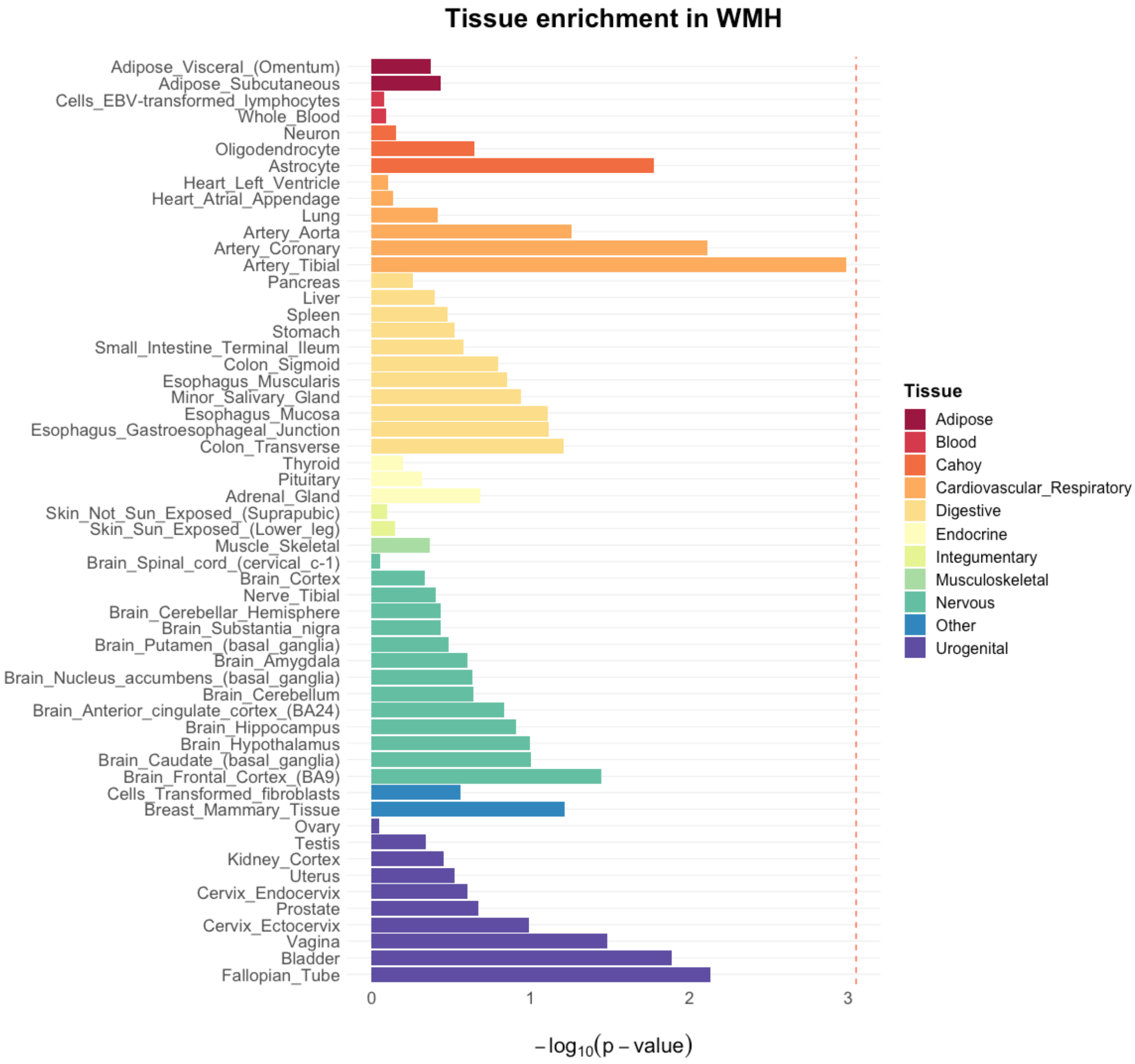

Supplementary Figure 11. Tissue enrichment in WMH GWAS meta-analysis summary statistics.

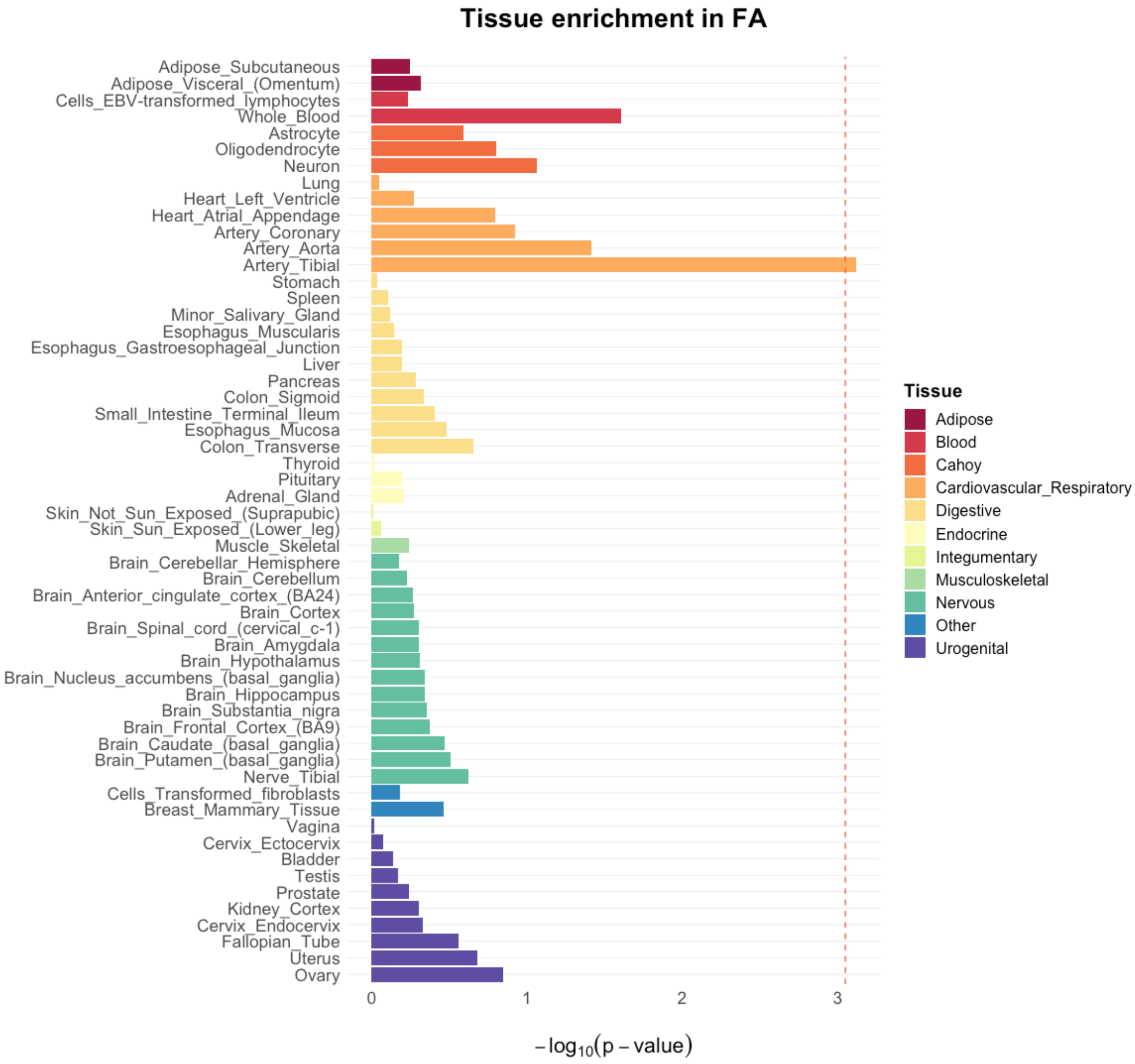

Supplementary Figure 12. Tissue enrichment in FA GWAS summary statistics.

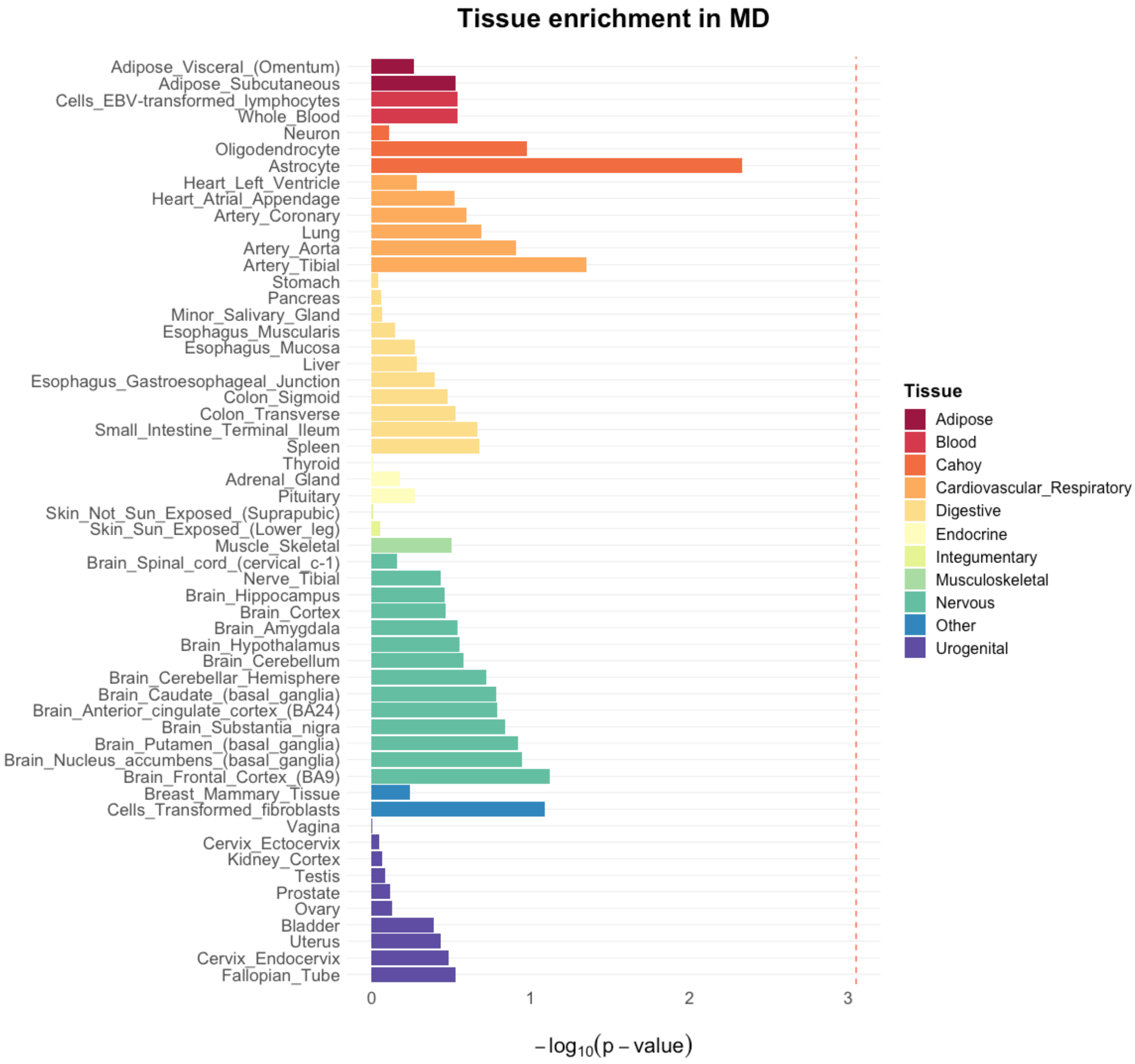

Supplementary Figure 13. Tissue enrichment in MD GWAS summary statistics.

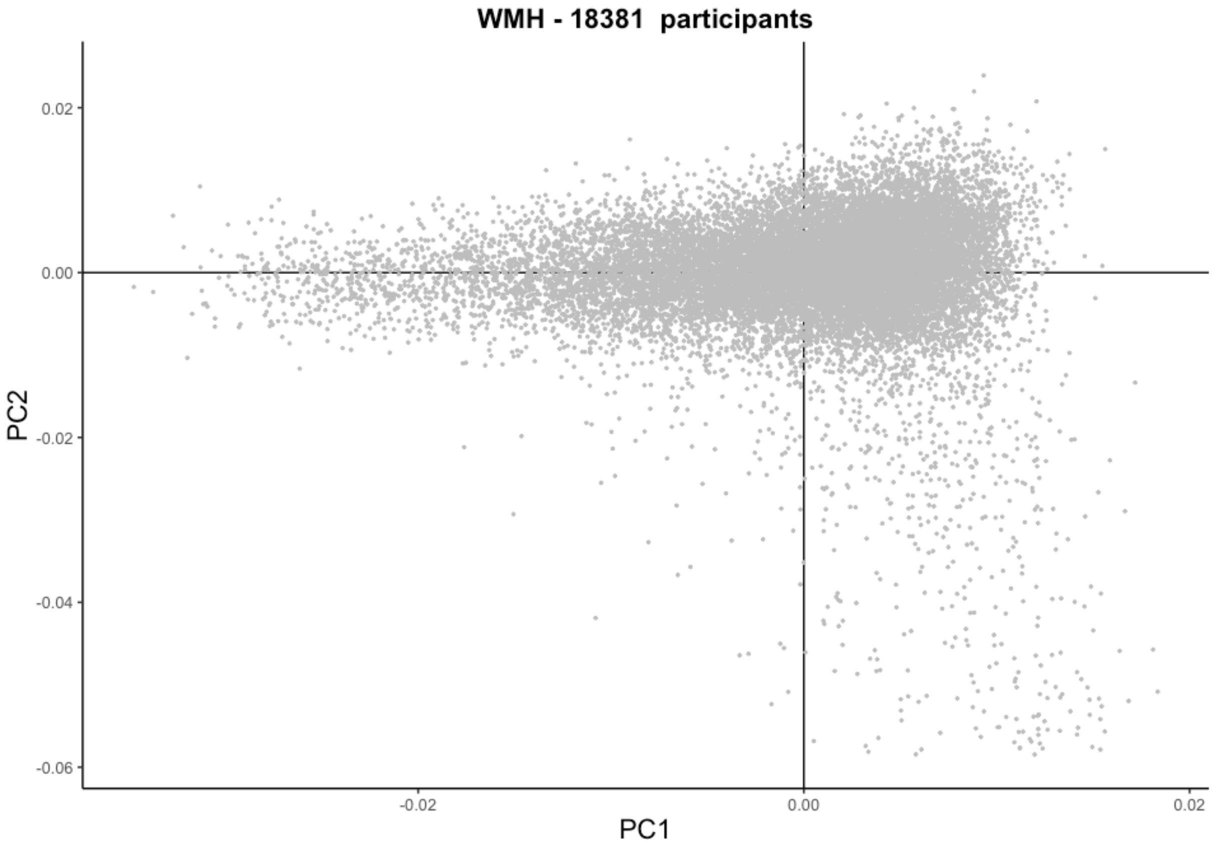

Supplementary Figure 14. Graph of individuals for PCA on WMH dataset.

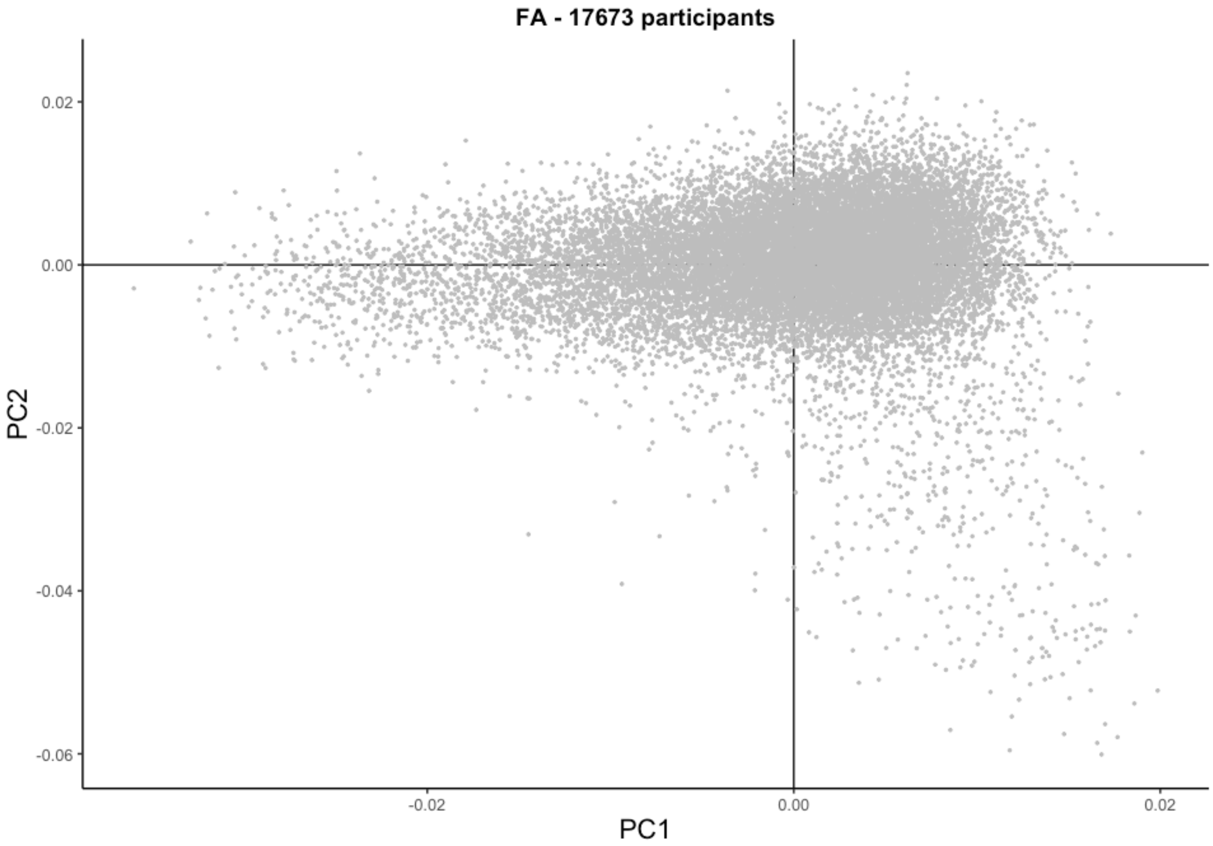

Supplementary Figure 15. Graph of individuals for PCA on FA dataset.

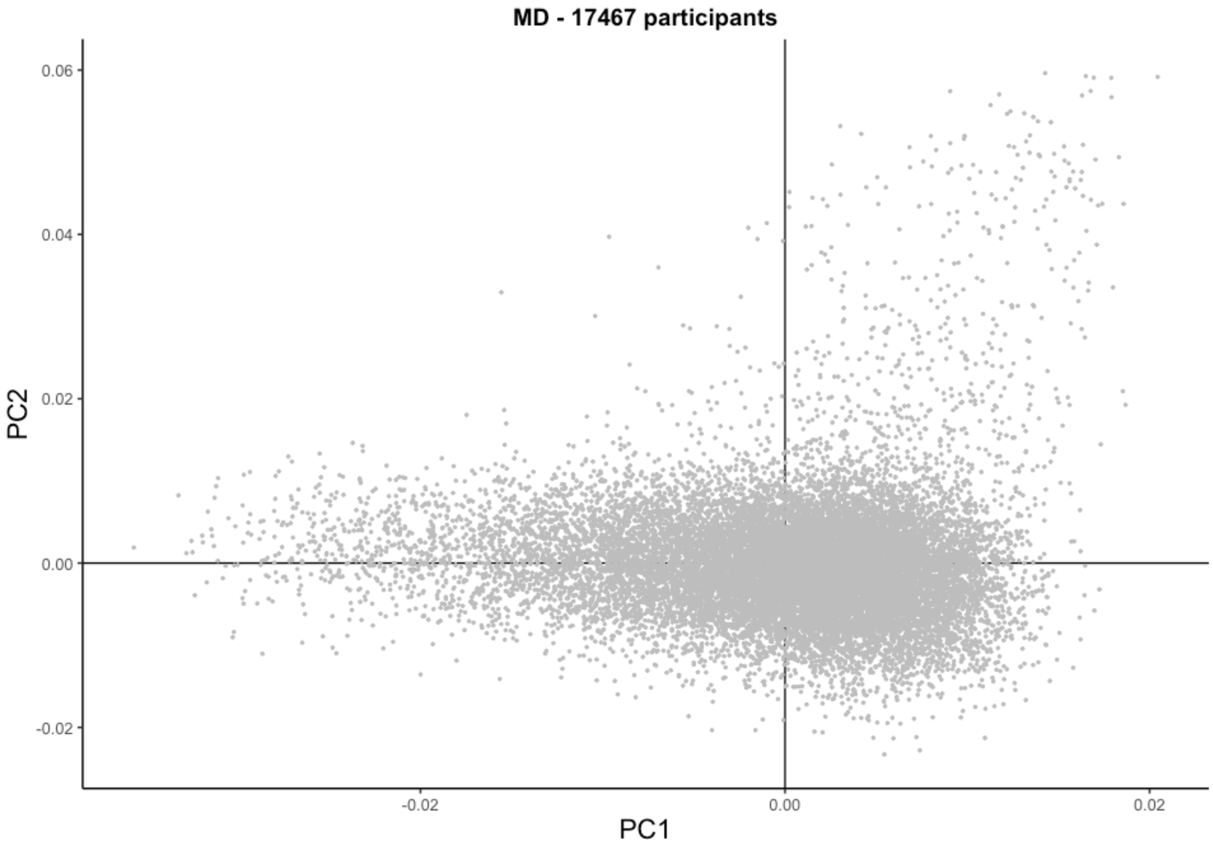

Supplementary Figure 16. Graph of individuals for PCA on MD dataset.

Supplementary Table 1. PCA eigenvalues and explained percentages of variance for FA and MD measure derivation

PC	Eigen value	Percentage of variance	Cumulative percentage of variance	Eigen value	Percentage of variance	Cumulative percentage of variance
1	18.17	37.86	37.86	19.57	40.76	40.76
2	3.7	7.71	45.58	4.86	10.13	50.9
3	2.62	5.46	51.04	3.58	7.47	58.36
4	1.86	3.87	54.91	2.4	5.01	63.37
5	1.72	3.59	58.5	2.13	4.43	67.8

Supplementary Table 2. GO enrichment analysis.

TRAIT	SET	NGENES	BETA	BETA_STD	SE	P	Name	Category	pBH
WMH	GO:0098910	3	2.31	0.0294	0.553	1.51E-05	regulation of atrial cardiac muscle cell action potential	biological process	0.04085
WMH	GO:0048050	3	2.39	0.0305	0.584	2.08E-05	post-embryonic eye morphogenesis	biological process	0.04085
WMH	GO:0005925	397	0.181	0.0263	0.0416	6.47E-06	focal adhesion	cell component	0.031746
WMH	GO:0031752	3	1.87	0.0239	0.405	1.95E-06	D5 dopamine receptor binding	molecular function	0.019137
WMH	GO:0016176	7	1.17	0.0228	0.285	2.05E-05	superoxide-generating NADPH oxidase activator activity	molecular function	0.04085
WMH	GO:0004713	81	0.363	0.024	0.0903	3.01E-05	protein tyrosine kinase activity	molecular function	0.049283
MD	GO:0098912	3	2.67	0.034	0.564	1.09E-06	membrane depolarization during atrial cardiac muscle cell action potential	biological process	0.003555
MD	GO:0086045	5	1.71	0.0281	0.415	1.99E-05	membrane depolarization during AV node cell action potential	biological process	0.048783
MD	GO:0086056	3	2.68	0.0341	0.543	4.09E-07	voltage-gated calcium channel activity involved in AV node cell action potential	molecular function	0.003555
MD	GO:0005540	24	0.77	0.0278	0.162	1.01E-06	hyaluronic acid binding	molecular function	0.003555

TRAIT: imaging trait; SET: gene set as the GO term ID; NGENES: the number of genes in the data that are in the set; BETA: the regression coefficient of the variable; BETA_STD: the semi-standardized regression coefficient; SE: the standard error of the regression coefficient; P: p-value for gene-set enrichment; Name: the GO term name; Category: the GO category; pBH: p-value after Benjamini-Hochberg FDR multiple testing correction.

Supplementary Table 3. Contribution of cardiovascular risk factors to WMH measure in UK Biobank population.

Risk factors	Field derivation	Sample size	Adjusted R2	P-value
Average systolic blood pressure, automated reading	mean(f4080_2_0,f4080_2_1)	15444	0.0108	1.34×10-38
Average diastolic blood pressure, automated reading	mean(f4079_2_0,f4079_2_1)	15444	0.0096	2.49×10-34
Waist/hip ratio	f48_2_0/f49_2_0	17979	0.0067	2.34×10-28
Average weekly alcohol intake	f1588_2_0 + f1578_2_0 + f1608_2_0 + f5364_2_0 + f1598_2_0	13226	0.0058	1.15×10-18
Body mass index	f21001_2_0	17926	0.0047	1.93×10-20
Diabetes diagnosed by doctor	f2443_2_0	18225	0.0039	2.48×10-17
Ever smoked	f20160_2_0	18197	0.0013	8.33×10-07

Supplementary Table 4. Mouse brain cell-type enrichment analysis.

GENE_SET_DEF	SET	NGENES	BETA	BETA_STD	SE	P
TOP100	aaSMC	91	0.136	0.00953	0.0852	0.054989
TOP100	AC	84	-0.0768	-0.00517	0.0897	0.80392
TOP100	aEC	115	0.0169	0.00133	0.0808	0.41737
TOP100	aSMC	95	0.15	0.0108	0.088	0.04386
TOP100	capilEC	102	-0.0576	-0.00427	0.0784	0.76877
TOP100	EC1	109	0.0904	0.00693	0.0827	0.13715
TOP100	EC2	110	-0.12	-0.00922	0.0793	0.93471
TOP100	EC3	95	0.0404	0.00289	0.0836	0.3142
TOP100	FB1	113	-0.16	-0.0125	0.0832	0.97263
TOP100	FB2	106	0.109	0.00825	0.0869	0.10442
TOP100	MG	117	-0.0472	-0.00374	0.0786	0.7258
TOP100	OL	97	0.0376	0.00272	0.0816	0.32244
TOP100	PC	102	-0.0551	-0.00408	0.08	0.75441
TOP100	vEC	89	0.0563	0.0039	0.0823	0.24676
TOP100	vSMC	89	-0.125	-0.00866	0.0896	0.91862
TOP500	aaSMC	535	0.0319	0.00535	0.037	0.19425
TOP500	AC	460	0.0322	0.00503	0.0396	0.20749
TOP500	aEC	545	0.0299	0.00506	0.0357	0.20154
TOP500	aSMC	528	0.0519	0.00864	0.0377	0.08441
TOP500	capilEC	519	0.0564	0.00931	0.0363	0.060042
TOP500	EC1	515	0.026	0.00427	0.0368	0.23995
TOP500	EC2	541	-0.0915	-0.0154	0.0355	0.99502
TOP500	EC3	526	0.0244	0.00406	0.0365	0.25171
TOP500	FB1	485	-0.00884	-0.00141	0.038	0.59196
TOP500	FB2	500	0.0759	0.0123	0.0391	0.026026
TOP500	MG	544	-0.0273	-0.00462	0.0365	0.77292
TOP500	OL	480	0.0522	0.0083	0.0378	0.083886
TOP500	PC	494	0.0124	0.002	0.0381	0.37261
TOP500	vEC	507	0.0129	0.00211	0.0368	0.36306
TOP500	vSMC	500	0.033	0.00535	0.0383	0.19469

Abbreviations: SMC: smooth muscle cell; EC: endothelial cell; PC: pericytes; vSMC: venous SMC; aaSMC: arteriolar SMC; aSMC: arterial SMC; MG: microglia; FB1: fibroblast-like type I; FB2: fibroblast-like type II; OL: oligodendrocytes; EC1: EC type 1; EC2: EC type 2; EC3: EC type 3; vEC: venous EC; capilEC: capillary EC; aEC: arterial EC; AC: astrocyte; GENE_SET_DEF: gene-set definition, i.e. top 100 or top 500 differentially expressed genes between the cell type vs other cell types; SET: cell-type gene set; NGENES: the number of genes in the data that are in the set; BETA: the regression coefficient of the variable; BETA_STD: the semi-standardized regression coefficient; SE: the standard error of the regression coefficient; P: p-value for gene-set enrichment

Supplementary Table 5. Removed field codes description.

	Non-cancer illness code, self-reported (20002)	ICD9 (41203, 41205)	ICD10 (41202, 41204)
**Stroke**	1081, 1086, 1491, 1583	430, 431, 434, 436	I60, I61, I63, I64
**Multiple sclerosis**	1261	340	G35
**Parkinson**	1262	332	G20
**Dementia**	1263	290	F00-F03
**Neurodegenrative**	1397	341	G30-G32, G36, G37

Supplementary Table 6. Sample quality control description.

	WMH	FA	MD
**Individuals with phenotype data**	20,200	19,395	19,395
**With a disease code**	-409	-398	-398
**Remaining**	19,791	18,997	18,997
**Outliers**	0	-71	-282
**Remaining**	19,791	18,926	18,715
**No QC information**	-508	-471	-260
**Remaining**	19,283	18,455	18,455
**(1) Kinship >= 0.0884**	-156	-145	-144
**(2) Gender mismatch**	-15	-14	-13
**(3) Outliers (het. missing.)**	-37	-36	-36
**(3) Missing rate > 0.05**	-14	-14	-14
**(4) No European ancestry**	-481	-455	-449
**Intersect from (1) to (4)**	-688	-649	-641
**Remaining**	18,595	17,806	17,605
**Outliers with smartpca**	-213	-143	-138
**Individuals for GWAS analysis**	18,381	17,663	17,467

Supplementary Table 7. UK Biobank field codes to derive covariates.

Covariate	Field code	Description
**Sex**	31	Sex
**Age at MRI**	34	Year of birth
	52	Month of birth
	53	Date of attending assessment centre
**UKB centre**	54	UK Biobank assessment centre
**Genotype Batch**	Note: From sample QC file	
**Mean rfMRI**	25741	Mean rfMRI head motion, averaged across space and time points
**Mean rfMRI**	25742	Mean tfMRI head motion, averaged across space and time points
**PC1 – PC10**	Note: Derived from PCA on genotype data	

Supplementary Table 8. Covariate description.

Covariate		WMH	FA	MD
		N = 18,381	N = 17,673	N = 17,467
Sex	Female	9,688 (52.7%)	9,362 (53.0%)	9,278 (53,1%)
	Male	8,693 (47.3%)	8,311 (47.0%)	8,189 (46.9%)
Age at MRI	Mean (Sd)	63.3 (7.4)	63.2 (7.4)	63.1 (7.4)
UKB centre	Cheadle	15,431 (84.0%)	14,802 (83.8%)	14,622 (83.7%)
	Newcastle	2,950 (16.0%)	2,871 (16.2%)	2,845 (16.3%)
Genotype Batch	UKBB	16,582 (90.2%)	15,943 (90.2%)	15,759 (90.2%)
	UKBL	1,799 (9.8%)	1,730 (9.8%)	1,708 (9.8%)
Mean rfMRI	Number of missing values	799	58	57
Mean tfMRI	Number of missing values	1847	1109	1095
PC1-PC10				

Supplementary Table 9. Description of covariates in relation to WMH, FA and MD phenotypes

Covariate			WMH	FA	MD
			N = 18,381	N = 17,673	N = 17,467
Sex	Female	Mean (Sd)	7.80 (1.15)	-0.14 (4.2)	-0.38 (4.33)
	Male	Mean (Sd)	8.13 (1.18)	0.16 (4.33)	0.43 (4.48)
Age at MRI	r2		0.6	-0.35	0.4
UKB centre	Cheadle	Mean (Sd)	7.91 (1.18)	0.09 (4.24)	0.19 (4.39)
	Newcastle	Mean (Sd)	8.21 (1.12)	-0.47 (4.35)	-0.96 (4.45)
Genotype Batch	UKBB	Mean (Sd)	7.95 (1.18)	0.01 (4.26)	-0.01 (4.42)
	UKBL	Mean (Sd)	7.98 (1.20)	-0.11 (4.30)	0.06 (4.50)
Mean rfMRI	r2		0.23	-0.15	0.11
Mean rfMRI	r2		0.27	-0.18	0.14
PC1	r2		-0.019	-0.008	0.037
PC2	r2		0.006	-0.01	-0.035
PC3	r2		0.03	0.01	0.006
PC4	r2		0	0.001	-0.008
PC5	r2		0.007	-0.004	0.011
PC6	r2		-0.006	0.008	-0.008
PC7	r2		0.003	-0.004	0
PC8	r2		0.007	0.005	0.005
PC9	r2		0.005	0.007	-0.012
PC10	r2		0.007	-0.008	0.004
