# Supplementary material for: Genome-wide association study of MRI markers of cerebral small vessel disease in 42,310 participants
Source: Nat Commun. 2020 May 1;11:2175. doi: 10.1038/s41467-020-15932-3 (PMC7195435; doi:10.1038/s41467-020-15932-3)
Supplement: Supplementary file 11 — Reporting Summary [file 41467_2020_15932_MOESM11_ESM.pdf]

nature research

Corresponding author(s): Hugh Markus

Last updated by author(s): Mar 13, 2020

Reporting Summary

Nature Research wishes to improve the reproducibility of the work that we publish. This form provides structure for consistency and transparency in reporting. For further information on Nature Research policies, see Authors & Referees and the Editorial Policy Checklist .

Statistics

For all statistical analyses, confirm that the following items are present in the figure legend, table legend, main text, or Methods section.

n/a	Confirmed	
☐	☒	The exact sample size (*n*) for each experimental group/condition, given as a discrete number and unit of measurement
☐	☒	A statement on whether measurements were taken from distinct samples or whether the same sample was measured repeatedly
☒	☐	The statistical test(s) used AND whether they are one- or two-sided<br>*Only common tests should be described solely by name; describe more complex techniques in the Methods section.*
☐	☒	A description of all covariates tested
☐	☒	A description of any assumptions or corrections, such as tests of normality and adjustment for multiple comparisons
☐	☒	A full description of the statistical parameters including central tendency (e.g. means) or other basic estimates (e.g. regression coefficient) AND variation (e.g. standard deviation) or associated estimates of uncertainty (e.g. confidence intervals)
☐	☒	For null hypothesis testing, the test statistic (e.g. *F*, *t*, *r*) with confidence intervals, effect sizes, degrees of freedom and *P* value noted<br>*Give P values as exact values whenever suitable.*
☒	☐	For Bayesian analysis, information on the choice of priors and Markov chain Monte Carlo settings
☒	☐	For hierarchical and complex designs, identification of the appropriate level for tests and full reporting of outcomes
☐	☒	Estimates of effect sizes (e.g. Cohen's *d*, Pearson's *r*), indicating how they were calculated

Our web collection on statistics for biologists contains articles on many of the points above.

Software and code

Policy information about availability of computer code

Data collection	No code nor software was used for data collection.
Data analysis	FUSION 2018-08-01<br>LDSC 2017-02-05<br>MAGMA v1.06b<br>METAL 2011-03-25<br>PLINK v1.90b3.31<br>PLINK 2.0 2019-01-02<br>qctool v2.0<br>R package coloc v3.2.1<br>R package FactoMiner v1.42<br>R package hyprcoloc v1.0<br>R package mice v3.6.0<br>R package phenoscanner v1.0<br>TWAS-GSEA 2019-10-23<br><br>The code will be made publicly available on https://github.com/elodiepersyn before publication.

For manuscripts utilizing custom algorithms or software that are central to the research but not yet described in published literature, software must be made available to editors/reviewers. We strongly encourage code deposition in a community repository (e.g. GitHub). See the Nature Research guidelines for submitting code & software for further information.

Data

Policy information about availability of data

All manuscripts must include a data availability statement. This statement should provide the following information, where applicable:

- Accession codes, unique identifiers, or web links for publicly available datasets
- A list of figures that have associated raw data
- A description of any restrictions on data availability

This analysis used publicly available data from the UK Biobank (www.ukbiobank.ac.uk, field codes are described in the Supplementary Data 13 and the Supplementary Table 7), WMH stroke study (http://cerebrovascularportal.org/informational/downloads) and CHARGE (https://www.ncbi.nlm.nih.gov/gap/, we used data from the study phs000930.v6.p1, the currently available version is phs000930.v7.p1). The GWAS summary statistics from WMH, FA and MD for the UK Biobank and stroke studies are available via the ISGC cerebrovascular disease knowledge portal (http://www.cerebrovascularportal.org/informational/data). We obtained the CHARGE summary statistic data directly from dbGaP. We are unable to make them available via the cerebrovascular disease portal due to dbGaP and CHARGE access regulations, and these can be obtained direct from dbGaP (https://www.ncbi.nlm.nih.gov/gap/). In our post-GWAS analyses, we used the Gene Ontology database (http://geneontology.org/), MAGMA software gene definitions (https://ctg.cncr.nl/software/magma), the PhenoScanner database (http://www.phenoscanner.medschl.cam.ac.uk/), LDSC LD scores (https://github.com/bulik/ldsc), GWAS summary statistics (the list of Pubmed IDs is provided in the Supplementary Data 5), FUSION software weights and reference LD (http://gusevlab.org/projects/fusion/), differential expression data in mouse brain cell types (http://betsholtzlab.org/VascularSingleCells/database.html).

Field-specific reporting

Please select the one below that is the best fit for your research. If you are not sure, read the appropriate sections before making your selection.

[x] Life sciences [ ] Behavioural & social sciences [ ] Ecological, evolutionary & environmental sciences

For a reference copy of the document with all sections, see nature.com/documents/nr-reporting-summary-flat.pdf

Life sciences study design

All studies must disclose on these points even when the disclosure is negative.

Sample size	No statistical method was used to determine sample size. After quality control, the GWAS sample sizes in UK Biobank for white matter hyperintensity (WMH), fractional anisotropy (FA) and mean diffusivity (MD) were respectively 18,381, 17,663 and 17,467. We performed a meta-analysis for WMH with summary statistics from CHARGE study (N=21,079) and a study in stroke patients (N=2,850) for a total of 42,310 individuals.
Data exclusions	From UK Biobank participants, we excluded individuals with stroke, multiple sclerosis, Parkinson, dementia or neurodegenerative disease as in the previous GWAS study from Rutten-Jacobs et al. (2018).<br>For sample QC and SNP QC, we proceeded to a standard procedure with thresholds being set arbitrarily prior to the analyses.<br>The sample QC consisted in removing phenotypic outliers (outside the +/- 6 s.d. range), individuals with no genotypic QC information, related individuals (kinship>=0.0884), gender mismatches, genotypic outliers in terms of heterozigosity and missingness, individuals with a genotype missing rate > 0.05, individuals from non-European ancestry.<br>For the SNP QC, we removed from the analysis variants with an imputation INFO score <0.5, a MAF<1% or a Hardy-Weinberg disequilibrium p-value < 1e-10.
Replication	We did not proceed to the replication of our findings as we wanted to increase the power of our meta-analysis by gathering a maximum number of participants.
Randomization	No participants were randomized in this study as we are analyzing observational data.
Blinding	We used datasets from UK Biobank, CHARGE and a study in stroke patients, which were collected independently from the association analyses.

Reporting for specific materials, systems and methods

We require information from authors about some types of materials, experimental systems and methods used in many studies. Here, indicate whether each material, system or method listed is relevant to your study. If you are not sure if a list item applies to your research, read the appropriate section before selecting a response.

Materials & experimental systems

n/a	Involved in the study
☒	☐ Antibodies
☒	☐ Eukaryotic cell lines
☒	☐ Palaeontology
☒	☐ Animals and other organisms
☐	☒ Human research participants
☒	☐ Clinical data

Methods

n/a	Involved in the study
☒	☐ ChIP-seq
☒	☐ Flow cytometry
☒	☐ MRI-based neuroimaging

Human research participants

Policy information about studies involving human research participants

Population characteristics	The UK Biobank population for the WMH trait included 52.7% female and 47.3% male European participants. The average age at MRI scan was 63.3 years old (sd 7.4). MRI data was collected in two imaging centres, 84% from Cheadle UKB centre and 16% from the Newcastle UKB centre. The UK Biobank population characteristics for the two other imaging biomarkers FA and MD were very similar as including almost all the same participants who underwent MRI scan.<br>For the WMH meta-analysis we used summary statistics from CHARGE study (Verhaaren et al., 2015) and a study in stroke patients (Traylor et al., 2019), the population characteristics being described in the original papers.
Recruitment	UK Biobank recruited volunteer participants from the UK population to gather a major health resource. CHARGE consortium gathered data for participants from different health cohorts worldwide. For the WMH study in ischemic stroke patients, participants were recruited through hospital-based studies.
Ethics oversight	This research has been conducted using the UK Biobank Resource under application number 36509. UK Biobank received ethical approval from the Research Ethics Committee (reference 16/NW/0274). CHARGE summary statistics were obtained through the dbGaP portal application number 19896 (study: phs000930.v6.p1). Summary statistics from the WMH study in stroke patients were obtained through agreement with the authors 7. All studies obtained informed consent from all participants and got ethical approval from their local ethics committee; full ethical permissions of contributing studies have been previously published.

Note that full information on the approval of the study protocol must also be provided in the manuscript.
