# Supplementary material for: Genome-wide association study of MRI markers of cerebral small vessel disease in 42,310 participants
Source: Nat Commun. 2020 May 1;11:2175. doi: 10.1038/s41467-020-15932-3 (PMC7195435; doi:10.1038/s41467-020-15932-3)
Supplement: Supplementary file 12 — Description of Additional Supplementary Files [file 41467_2020_15932_MOESM12_ESM.pdf]

Title: Supplementary Data 1.
Description: GWAS meta-analysis association loci for WMH.

Title: Supplementary Data 2.
Description: GWAS association results for FA.

Title: Supplementary Data 3.
Description: GWAS association results for MD.

Title: Supplementary Data 4.
Description: Genetic correlation results for WMH, FA and MD.

Title: Supplementary Data 5.
Description: Significant genetic correlations with and without stroke cases from the third GWAS study.

Title: Supplementary Data 6.
Description: HyprColoc results.

Title: Supplementary Data 7.
Description: PhenoScanner annotations.

Title: Supplementary Data 8.
Description: TWAS associations results for WMH, FA and MD.

Title: Supplementary Data 9.
Description: UK Biobank field codes to derive output phenotypes
